# Modelling aggressive prostate cancers of young men in immune-competent mice, driven by isogenic *Trp53* alterations and *Pten* loss

**DOI:** 10.1038/s41419-022-05211-y

**Published:** 2022-09-08

**Authors:** Javier Octavio Mejía-Hernández, Simon P. Keam, Reem Saleh, Fenella Muntz, Stephen B. Fox, David Byrne, Arielle Kogan, Lokman Pang, Jennifer Huynh, Cassandra Litchfield, Franco Caramia, Guillermina Lozano, Hua He, James M. You, Shahneen Sandhu, Scott G. Williams, Ygal Haupt, Sue Haupt

**Affiliations:** 1 Peter MacCallum Cancer Centre, 305 Grattan St, Melbourne, VIC 3000 Australia; 2 Sir Peter MacCallum Department of Oncology, The University of Melbourne, Parkville, VIC 3010 Australia; 3 Tumour Suppression and Cancer Sex Disparity Laboratory, Peter MacCallum Cancer Centre, 305 Grattan St, Melbourne, VIC 3000 Australia; 4 Pathology Department, Peter MacCallum Cancer Centre, 305 Grattan St, Melbourne, VIC 3000 Australia; 5 Olivia Newton-John Cancer Research Institute, School of Cancer Medicine, La Trobe University, Heidelberg, VIC 3084 Australia; 6 Department of Genetics, The University of Texas MD Anderson Cancer Center, Houston, TX USA; 7 University of Texas MD Anderson Cancer Center UTHealth Graduate School of Biomedical Sciences, University of Texas, Houston, TX USA; 8 Department of Hematopathology, UT MD Anderson Cancer Center, Houston, TX USA; 9 Department of Medical Oncology, Peter MacCallum Cancer Centre, Parkville, VIC 3000 Australia; 10 Division of Radiation Oncology, Peter MacCallum Cancer Centre, 305 Grattan St, Melbourne, VIC 3000 Australia; 11 Present Address: Telix Pharmaceuticals Ltd, Melbourne, VIC 3051 Australia; 12 Present Address: CSL Innovation, CSL Ltd, Melbourne, VIC 3052 Australia; 13 Present Address: Vittail Ltd, Melbourne, VIC 3146 Australia

**Keywords:** Cancer models, Experimental models of disease

## Abstract

Understanding prostate cancer onset and progression in order to rationally treat this disease has been critically limited by a dire lack of relevant pre-clinical animal models. We have generated a set of genetically engineered mice that mimic human prostate cancer, initiated from the gland epithelia. We chose driver gene mutations that are specifically relevant to cancers of young men, where aggressive disease poses accentuated survival risks. An outstanding advantage of our models are their intact repertoires of immune cells. These mice provide invaluable insight into the importance of immune responses in prostate cancer and offer scope for studying treatments, including immunotherapies. Our prostate cancer models strongly support the role of tumour suppressor p53 in functioning to critically restrain the emergence of cancer pathways that drive cell cycle progression; alter metabolism and vasculature to fuel tumour growth; and mediate epithelial to mesenchymal-transition, as vital to invasion. Importantly, we also discovered that the type of p53 alteration dictates the specific immune cell profiles most significantly disrupted, in a temporal manner, with ramifications for disease progression. These new orthotopic mouse models demonstrate that each of the isogenic hotspot p53 amino acid mutations studied (R172H and R245W, the mouse equivalents of human R175H and R248W respectively), drive unique cellular changes affecting pathways of proliferation and immunity. Our findings support the hypothesis that individual p53 mutations confer their own particular oncogenic gain of function in prostate cancer.

## Background

Prostate cancer (PC) is the most frequently diagnosed male cancer and among the top three leading causes of male cancer death in extensive world populations, including the United States of America, Europe [1], China [2] and Australia [3]. The overwhelming majority of men with PC are successfully treated (~90%; [4–6]) when the disease is confined to the primary site. For an unfortunate minority of ~10%, PC spreads outside the prostate glands and invades distant sites. The treatment options for men with PC that has metastasised to remote secondary sites remains limited. Around 70% of patients with metastatic PC do not live more than five-years [7], due to the development of metastatic castrate resistant PC (mCRPC [5]).

There has been a resounding failure of the new wave of immunotherapies to impact PC for most patients trialled. Consistently, PCs are notoriously poorly infiltrated by immune cells, resulting in inadequate immune surveillance and deficient clearance of PC cells [8]. The causes of ineffective immune responses in most PC patients are not fully understood and uncovering the explanations are expected to expose strategic therapeutic approaches to countering these deficiencies.

A rational approach to guide intensified treatment for aggressive mCRPC, is to discover its drivers and use these as predictive molecular biomarkers in advance of spreading. Molecular studies are at the core of extensive ongoing PC research (e.g. [9, 10]). Outstanding among the few robust biomarkers of PC metastasis is mutation of the major tumour suppressor protein p53 [9, 11, 12]. *TP53* mutation was identified in up to ~73% of lethal metastatic PCs (reviewed [13]). Consistently, clustering of p53 positive cells in primary tumours from radical prostatectomies proved reliably predictive of metastatic relapse and PC-specific death [14].

p53 has also recently been linked to altered immunity (as we reviewed [15]). How p53 impacts immune infiltration in PC specifically, and in turn affects spread from the primary tumour site and influences therapeutic responses, are open and pertinent questions. Notably, p53 mutation alone is insufficient to cause full scale PC and requires accompanying driver mutations. A potent additional event is the loss of the tumour suppressor PTEN (Phosphatase and tensin homologue). *PTEN* loss occurs in ~20% of primary PC and increases to ~40–60% in mCRPC [9]. *PTEN* deletion has also been linked to altered immune cell profiles [16] and immunosuppressive environments in PC [17]. The combined alteration of *PTEN* and *TP53*, is frequent in mCRPC (reviewed in [18]).

Development of pre-clinical models that translate to human immunity, is ranked number one, among ten key challenges recently identified for developing effective cancer immunotherapy. Number two on the list is the identification of the dominant drivers of cancer immunity [19]. Our studies are directed to address these challenges specifically in PC. Animal models with intact immune compartments provide physiological context that surpasses the value of cell lines and tumouroids/organoids [20]. The closer the model to emulating human PC, the greater its clinical relevance and this is our overriding motivation for developing advanced mouse models that are faithful to human disease.

In mice, loss of a single allele of *Pten* is capable of producing prostatic intraepithelial neoplasia (PIN). Loss of two *Pten* alleles drives slow advancement to local microinvasion. Metastatic prostate carcinoma is promoted when *Pten* loss co-occurs with other genetic alterations [21]. Combining *Pten* loss with *Trp53* alterations in mice is rational to faithful modelling of human disease and has been actively attempted (reviewed [22]; and here we note the distinction between the mouse and human gene names and use each as appropriate to the particular species: *Pten*: *PTEN*; *Trp53*: *TP53* respectively). Early mouse PC models used probascin (Pb)–driven Cre recombinase (Cre) to simultaneously knock-out (KO) *Pten* and *Trp53* genes from the prostate. An advanced model with a Cre-activatable luciferase reporter enabled live imaging of disease development. Tumours developed with a sarcomatoid histology and invaded locally to the peritoneal cavity, which did not spread to lymph nodes or distant sites [23]. While these models have the advantage of orthotopic development and are not immune compromised (in contrast to xenotransplants, for example of patient derived xenografts), they are severely limited by an inadequate modelling of the nature of the driver mutation; where mCRPC is frequently mediated by *TP53* missense mutation, rather than its loss. This deficiency defines a crucial gap-in-knowledge and led us to develop a new approach to designing an appropriate PC model.

In this study, we describe new models of human PC in mice that are constructed in an intact immune environment, which are driven by altered *Trp53* (either deletion or mutation of amino acids R172H or R245W) and *Pten* loss. We demonstrate that human PC can be faithfully recapitulated in mice by mimicking these major human alterations and reveal how they offer insight into the onset of the disease. These models show the relevance of the immune disruption, with scope for testing treatments, including precision and immune therapies against PCs. Human PC recapitulation in these mice models has led us to an advanced understanding of genetic and molecular alterations driving this most commonly diagnosed cancer in men.

## Results

### TP53 mutations differ in prevalence in human prostate cancer with age

**Fig. 1:**
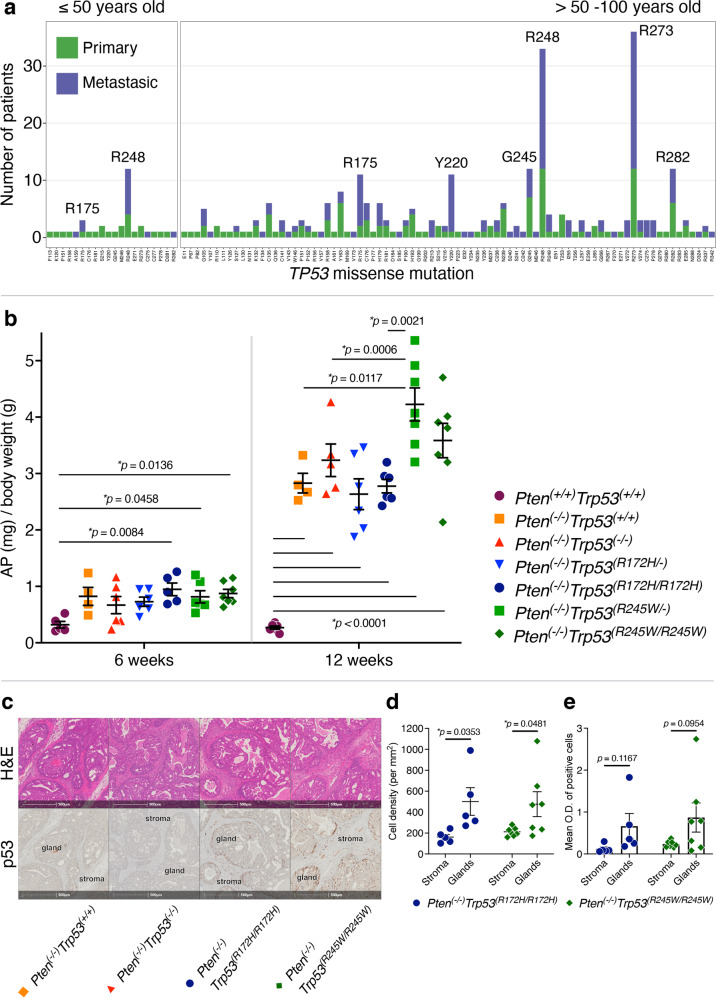
R175 and R248 are the most prevalent missense *TP53* mutations in ≤50 years old prostate cancer patients and are modelled by mouse isogenic *Trp53* mutant homologues (R172H and R245W). **a** Distribution of *TP53* missense mutations in primary and metastatic PC samples from patients ≤50 years of age, and >50–100 years (source Catalogue Of Somatic Mutations In Cancer). **b** Mean weights (mg) of anterior prostate (AP) lobes normalised to total body weight of 6 weeks-old and 12 weeks-old mice isogenic for *Trp53*. ANOVA and Tukey’s statistical tests were applied to calculate *p*-value significance between genotypes. Error bars indicate mean ± SEM (*n* = 4–7 mice). **c** Haematoxylin and eosin (H&E) and serially-matched p53 immunohistochemical staining of AP sections of 12-week old mice of indicated genotypes. High resolution-whole tissue scans were uploaded into HALO® and analysed for p53. **d** Quantification of p53^+^ cell density (per mm^2^) and **e** p53 mean optical density (O.D.) in two tissue zones (AP stroma and AP glands). *p*-values were calculated using a Two-tailed Student’s *t*-test. Error bars indicate mean ± SEM (*n* = 5–6 mice).

In PC, as with other cancers, *TP53* gene mutations are largely missense and located predominantly within the DNA Binding Domain (DBD) of the protein [24]. Individual p53 mutations confer distinct properties [25]. Accurate modelling of human PC in mice, relies on the adoption of relevant p53 mutations. To identify the frequency of *TP53* mutations in human PC, we analysed the Catalogue of Somatic Mutations in Cancer (COSMIC) database, which is the most extensive and comprehensive international collection of cancer somatic mutation data [26]. An intriguing finding from our analysis was that age stratifies PC mutation prevalence (Fig. 1a). Strikingly, we found that at younger age (≤50 years), the most frequent and also metastatic *TP53* mutations occurred at amino acids R248 and R175. In contrast, in PC patients >50 years, amino acid residue R273 was most commonly mutated.

### PC mice models isogenic for Trp53 mutations develop distinct disease phenotypes

Informed by our findings in young men with PC, we bred new mouse models with tissue-restricted disease induction, localised in the prostate gland epithelium. In this layer, the androgen-dependent Pb-Cre drove the alteration of the two key tumour suppressors *Pten* [9] and *Trp53* [13], emulating primary events relevant to human PC (see methods for explicit details). For simplicity, we will refer to the *Trp53* variants as: *Trp53*^*(+/+)*^, *Trp53*^*(−/−)*^, *Trp53*^*(R172H)*^ and *Trp53*^*(R245W)*^ (where the human equivalent of the missense mutants are *TP53*^*(R175H)*^ and *TP53*^*(R248W)*^, respectively). Under the influence of increasing androgen levels during pubescence (peaking around 5–6 weeks [27]), the age-dependent induction of *Pten* deletion and alterations in *Trp53*, either from wt to mutant (see methods) or its deletion, was designed to mimic human increased risk associated with somatic PC, rather than familial PC.

The rationale for homozygous *TP53* gene mutations was based on its high frequency (~50–60%) in human cancers, as reported in a study of the most comprehensive cancer data sets, collated in COSMIC [28]. Notably, *TP53* loss of heterozygosity (LOH) is extremely common in aggressive human cancers, as supported by >92% of tumours harbouring a single *TP53* mutation and no accompanying wt *TP53* allele [24].

*TP53* loss of heterozygosity (LOH) was found to be extremely common in aggressive human cancers in an extensive study of The Cancer Genome Atlas (TCGA) [24]. Among cancers with a single *TP53* mutation, >90% had lost their wt *TP53* allele. We modelled two scenarios consistent with *TP53* LOH. The first case simulated single *TP53* mutations existing in a homozygous diploid state. In humans these appear to arise through duplication of a *TP53* mutation, likely by mitotic recombination or another gene duplication mechanism, and accompanied by loss of the additional wt allele. The second case modelled conconimant hemizygous *TP53* mutation and accompanying loss of the wt allele. This is relevant to ~2/3 of cancers in TCGA with a single *TP53* mutation [24]. These data support the hypothesis that p53 functions as a classic recessive tumour suppressor, in which a consecutive two-hit mechanism typically involves a missense mutation in one allele, followed by loss of the second wt-p53 allele [24].

Adding to the biological importance of *TP53* LOH, is the finding that it is a pre-requisite for mutant p53 stabilisation and the acquisition of oncogenic activities, referred to as gain-of-functions (GOFs) in *vivo* [29]. This aligns with the demonstration that mouse mesenchymal stem cells with p53^(*R175H/+*)^ formed subcutaneous tumours upon transplantation only after undergoing LOH [30]. Upon this evidence, our four isogenic mouse models provide an unparalled context for faithful modelling of *TP53* Loss Of Functions (LOFs), versus mutant TP53 GOFs. For comparison, we also generated mice deleted for *Pten* that were either homozygous for wt *Trp53* or *Trp53* deletion. We also included control mice that were wt for *Pten* and *Trp53*.

To phenotype these isogenic mice we undertook terminal analyses at designated ages. At 6 weeks of age, loss of *Pten* emerged as the outstanding cause of tumour weight increase, restricted to the anterior prostate lobes (AP) and appeared independent of *Trp53* mutation/loss status (Fig. 1b). Notably, a rapid surge in circulating testosterone levels in male mice has been measured by others, starting from ~4 weeks of age, with the peak at ~5–6 weeks, after which, levels stabilise but remain relatively high [27]. Our observation of emerging pathology in the mice AP at 6 weeks of age is consistent with the reported early temporal increase in testosterone levels, driving localised expression of Cre under the influence of its Pb promoter. The restricted disease manifestation likely reflects the levels of Cre expression and penetrance. The particular Pb promoter adopted in these studies is relatively weak [31], which explains the AP-restricted disease focus in the PC epithelium.

At 12 weeks, AP weight gains were accentuated for *Pten*^*(−/−)*^ cohorts relative to those of *Pten*^*(+/+)*^ (Fig. 1b). Beyond this, the mean AP tumour weights of both *Trp53*^*(R245W/R245W)*^ and *Trp53*^*(R245W/-)*^ cohorts were elevated relative to the other genotypes. This revealed a GOF at 12 weeks for *Trp53*^*(R245W)*^ tumours that had not emerged in the *Trp53*^*(R172H)*^ counterparts. It is important to elaborate that up until 12 weeks, the weight gain in the prostate was measured to be restricted to the AP (as clear from Supplementary Fig. 1a, where the prostate weights subtracted of AP weights showed no significant weight differences between the genotypes).

It was pertinent to establish whether p53 protein levels differed between the genotypes, which could have been a potential contributor to distinct AP weights of the isogenic mutant mice at 12 weeks. To address this, we focused on the homozygous mice (Fig. 1c–e) and compared the relative density (Fig. 1d) and intensity (Fig. 1e) of p53 stained cells in the prostate gland epithelia and surrounding stroma. Importantly at 12 weeks, the p53 levels in the *Trp53*^*(R245W/R245W)*^ and *Trp53*^*(R172H/R172H)*^ AP lobes were similar, within comparable cellular regions of the AP. It is relevant to add that the cell density of p53 staining cells was higher in the glands than the stroma, consistent with human disease initiating in the glands. These data argue that AP weight differences at this time point cannot be dismissed as a result of discrepancy in levels of stabilised p53 protein between genotypes. This mouse data predict individuality between *Trp53* mutants in their acquisition of oncogenic functions: with *Trp53*^*(R245W)*^ being more aggressive than *Trp53*^*(R172H)*^. This aligns well with our data for human PCs (Fig. 1a), which identified more aggressive disease (associated with greater prevalence and higher metastatic burden) for *TP53*^*(R248)*^ than *TP53*^*(R175)*^. Another point worth adding, is that the 12 week time point is relevant to emerging PC in men, at a stage where patient surveillance is warranted.

**Fig. 2:**
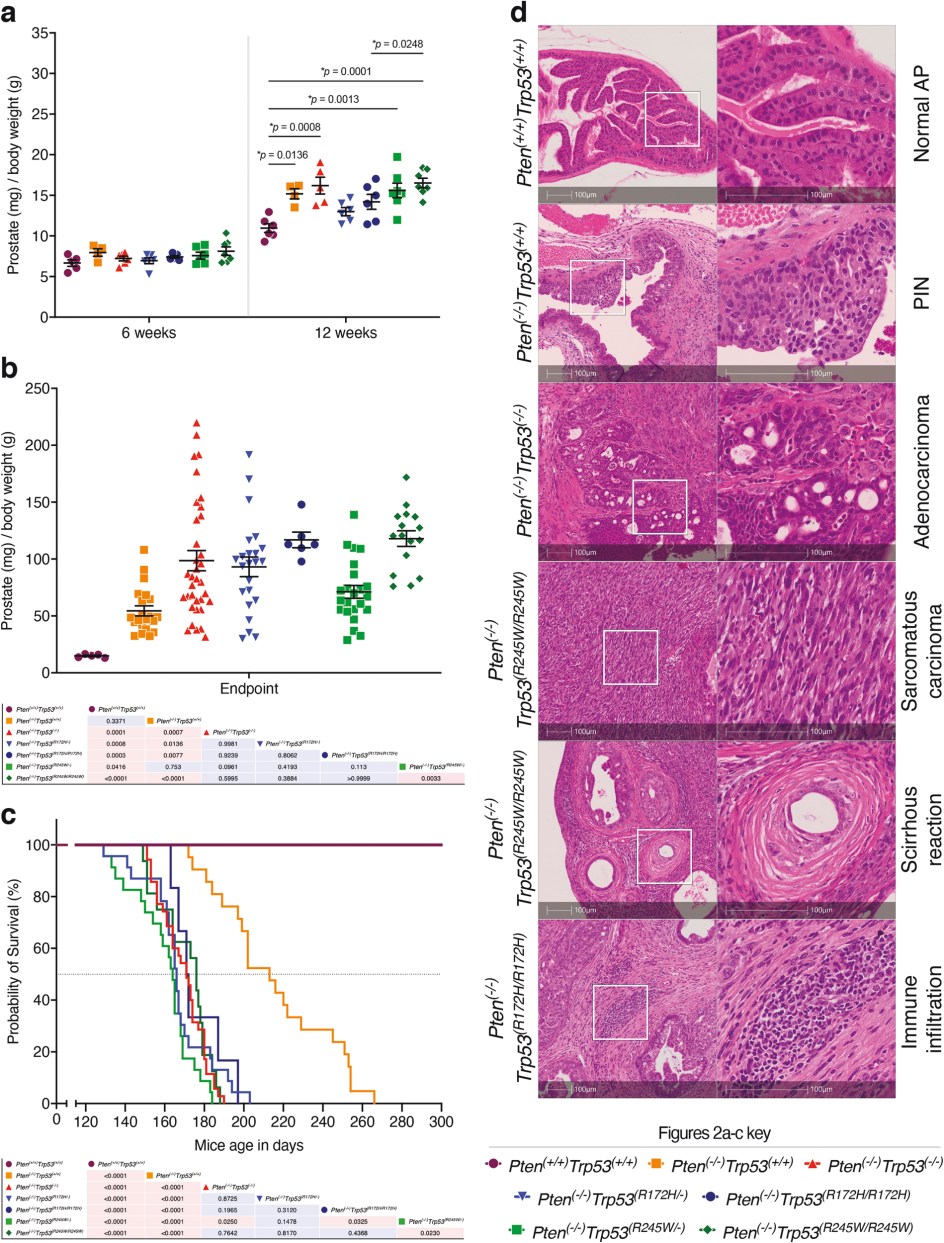
Tumour growth and aggressiveness is related to *Pten* haploinsufficiency and zygosity of distinct *Trp53* mutations. Mean weights (mg) of prostates dissected *en bloc* normalised to total body weight at: **a** 6 weeks-old, 12 weeks-old mice; and **b** at ethical endpoint for mice of indicated genotypes. Matrix showing *p*-values of prostate comparison at ethical endpoint; blue indicates *p*-value > 0.05 whereas red indicates *p*-value < 0.05. *p*-values resulted from an ANOVA and Tukey’s test. Error bars indicates mean ± SEM. Six weeks-old and 12 weeks-old mice *n* = 4–7. Endpoint comparisons *Pten*^*(+/+)*^*Trp53*^*(+/+)*^ *n* = 5; *Pten*^*(−/−)*^*Trp53*^*(+/+)*^ *n* = 21; *Pten*^*(−/−)*^*Trp53*^*(−/−)*^ *n* = 35; *Pten*^*(−/−)*^*Trp53*^*(R172H/−)*^ *n* = 23; *Pten*^*(−/−)*^*Trp53*^*(R172H/R172H)*^ *n* = 6; *Pten*^*(−/−)*^*Trp53*^*(R245W/−)*^ *n* = 23; *Pten*^*(−/−)*^*Trp53*^*(R245W/R245W)*^ *n* = 16. **c** Kaplan–Meier plot for mice of indicated genotypes as a function of survival probability and age. Statistical significance shown in the matrix below (blue indicates *p*-value > 0.05 whereas red indicates *p*-value < 0.05), result of Log-rank (Mantel–Cox) test. *Pten*^*(+/+)*^*Trp53*^*(+/+)*^ *n* = 5; *Pten*^*(−/−)*^*Trp53*^*(+/+)*^ *n* = 21; *Pten*^*(−/−)*^*Trp53*^*(−/−)*^ *n* = 35; *Pten*^*(−/−)*^*Trp53*^*(R172H/−)*^ *n* = 23; *Pten*^*(−/−)*^*Trp53*^*(R172H/R172H)*^ *n* = 6; *Pten*^*(−/−)*^*Trp53*^*(R245W/−)*^ *n* = 23; *Pten*^*(−/−)*^*Trp53*^*(R245W/R245W)*^ *n* = 16. **d** Representative and gross histopathological characteristics revealed by haematoxylin and eosin (H&E) staining of anterior prostate (AP) tissue at ethical endpoint. All mice showed morphological heterogeneity containing areas of prostate intraepithelial neoplasia (PIN), adenocarcinoma, sarcomatous carcinoma and scirrhous reaction. Inflammation was also observed as immune cell infiltration.

At endpoint, in contrast to the 12 week prostates, invasive behaviour of the tumour cells led to a less clear separation of the individual prostate lobes (Supplementary Fig. 1b), preventing accurate weight determinations of individual APs. To enable a relevant temporal comparison for each genotype, whole prostates (inclusive of drained bladders) were consequently weighed at 6 weeks, 12 weeks and endpoint (Fig. 2a, b). At 6 weeks no significant changes across the genotypes were observed. From 12 weeks, the whole prostate weights of control *Pten*^*(+/+)*^ *Trp53*^*(+/+)*^ mice were becoming surpassed by gains of all other genotypes, which we identified to be due to AP weight gains exclusively (Fig. 1b and Supplementary Fig. 1a).

At endpoint, the greatest PC tumour weights were evident for *Trp53*^*(R245W/R245W)*^ and *Trp53*^*(R172H/R172H)*^ and this corresponded to their doubling times, which respectively were faster than all other cohorts (Supplementary Fig. 2a–f). The poorest survival outcomes were measured for all the *Trp53* alterations on a *Pten* null background, relative to the control mice *Pten*^*(−/−)*^*Trp53*^*(+/+)*^ and *Pten*^*(+/+)*^*Trp53*^*(+/+)*^ (Fig. 2c). It is worth noting that Kaplan–Meier survival analyses did not distinguish significant survival differences among cohorts with either *Trp53* loss or mutation accompanied by *Pten* loss. This aligns with previous studies by our team (G.Lozano) demonstrating that *Trp53* germline deletion results in equivalent survival outcomes to mutant *Trp53* mice, even though disease manifestation differed [32].

Overall, histological examination of all tumours with *Pten* deletion, revealed the appearance of prostatic intraepithelial neoplasia (PIN) at around 12 weeks within the AP and sparing the seminal vesicles at this stage (Fig. 1c). At endpoint, which typically occurred at 4.5–5 months in *Trp53* altered genotypes, PIN progression to adenocarcinoma was associated with nests of tumour cells infiltrating prostate glands from the main duct space (Fig. 2d). Features of epithelial to mesenchymal transitioned cell populations, spindle-like, sarcomatous carcinoma, arose in the endpoint tumours. Advanced tumours frequently infiltrated other prostate lobes (ventral/lateral and dorsal), with immune infiltration in some mice and distended seminal vesicles. Of relevance to phenotyping this model, these changes in pathology correlate with altered fertility of these mice and typically, breeding was most successful from 6–10 weeks of age, but not beyond.

Our findings define a window between 12–20 weeks, to be appropriate for modelling the transition of PIN to prostate adenocarcinoma, in the isogenic *Trp53* mutant mice. It is relevant to add that human PC is standardly re-sected at the stage of adenocarcinoma. Mouse endpoint disease would only correlate with untreated PC patients, as modern standard of care is to undertake early surveillance, which affords timely life-saving intervention for most patients [5]. We pursued endpoint data with this caveat in mind, with the hypothesis that disease manifestation in these mice may offer some insight into extremely advanced disease.

**Fig. 3:**
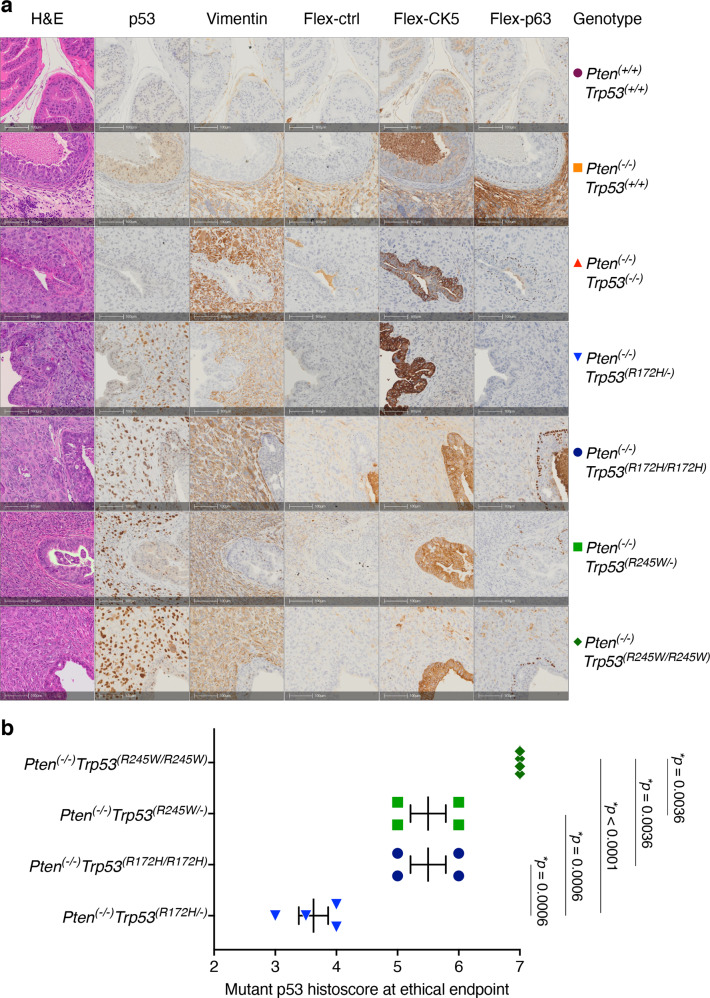
Immunohistochemical detection of cancer progression markers in anterior prostate lobes reveals characteristics displayed in aggressive human PC tumours. **a** Haematoxylin and eosin (H&E) staining and serially-matched immunohistochemical staining of sections of anterior prostate (AP) sections at ethical endpoint from mice of indicated genotype with antibodies against p53, vimentin, CK5 and p63. Flex-negative corresponds to CK5 and p63 immunohistochemical staining control. IHC staining was performed in >6 mice per genotype. Scale bar equivalent to 100 µm. **b** p53 staining intensity ranged from 0 (minimum) to 3 (maximum), whereas the proportion of cells stained was evaluated on a scale of 0 to 4 ((1) <10% stained; (2) 10–50% stained; (3) 50–80% stained; (4) >80%). Scores were summed to a final possible maximum histoscore of 7. *p*-values resulting from of statistical comparison of genotypes using ANOVA and Tukey’s tests. Error bars indicate mean ± SEM (*n* = 4 mice per genotype).

To characterise disease phenotypes in endpoint tumours, we used antibodies targeting clinically relevant antigens for analyses using immunohistochemistry (IHC) methods (Fig. 3a). Vimentin staining is indicative of epithelial to mesenchymal transition (EMT) and was evident in the tumour regions, while excluded from the glands that maintained normal architecture. Loss of Cytokeratin 5 (CK5) has been correlated with invasive adenocarcinoma, relative to PIN (as in a model with inducible loss of *Pten* and *Trp53* [33]). CK5 staining was the inverse of vimentin and was most pronounced in the normal-appearing glands. p63 is crucial for prostate development and is located in normal prostate basal cells but is not detected in human prostate adenocarcinomas [33]. In our studies, p63 was evident in end-stage tumours lacking *Pten* and maintaining wt *Trp53*. In the presence of mutant p53, p63 was less abundant (Fig. 3a). This is consistent with human studies demonstrating that mutant p53 inhibits expression of p63 and consequently abrogates the capacity of p63 to suppress EMT and ultimately metastasis [34].

To quantify differences in p53 staining at endpoint, histoscores were calculated as relevant to human pathology [35] (Histoscore = scaled percentage of positively p53 stained cells + p53 staining intensity). Notably, at endpoint, histoscores for p53 staining were highest for the heaviest tumours (Fig. 2b), with *Trp53*^*(R245W/R245W)*^ (7 = 4 + 3) surpassing *Trp53*^*(R172H/R172H)*^ (5 = 2.25 + 2.75); (Fig. 3b). It is relevant to emphasize that while the staining intensity was fairly comparable between these homozygous mutants (3:2.75), more cells stained positively among the *Trp53*^*(R245W/R245W)*^ tumours (4:2.25). Both elements of the histoscores for hemizygous mice were lower than their corresponding homozygous counterparts: *Trp53*^*(R245W/−)*^ (5.5 = 3 + 2.5); followed by *Trp53*^*(R172H/−)*^ (3.63 = 2.38 + 1.25). On note, there was quite a close correspondence between percentages of p53 stained cells and tumour doubling rates (Supplementary Fig. 2f).

Heterogeneity among tumour weights in the hemizygous mice, is likely to be explained by variation in the accumulation of fluid that we observed, although this was difficult to quantify (Supplementary Fig. 1b**)**. This fluid accumulation phenomenon was also evident in the *Trp53*^*(−/−*)^ tumours, where it also appeared to contribute heterogeneity among the weight gains measured (Fig. 2b). An interesting alignment appeared for the primary PC weights and those of the corresponding medial iliac lymph nodes, (Supplementary Fig. 3b–d), without evidence of metastatic tumour invasion. Such lymphadenopathy (nodal swelling), is interpreted as evidence of immune activation apparently triggered by the primary tumours and has been reported in men with PC adenocarcinoma [36].

In summary, in these genetically engineered isogenic models, PC initiated in the AP. The most rapid tumour growth was evident in the homozygous tumours. For subsequent analyses we chose to focus on tumours of *Trp53*^*(R245W/R245W)*^ and *Trp53*^*(R172H/R172H)*^ as they respectively displayed high cellular p53 protein levels, corresponding to the most rapid tumour doubling rates (Supplementary Fig. 2f) and endpoint tumour weights (Fig. 2b). As striking differences began to emerge at 12 weeks of age, with particular relevance to onsetting human disease, we studied this group primarily, in comparison to matched timepoint controls and also endpoint tumours.

### Isogenic *Trp53* tumours have temporally distinct immune profiles as measured by protein markers and gene expression

**Fig. 4:**
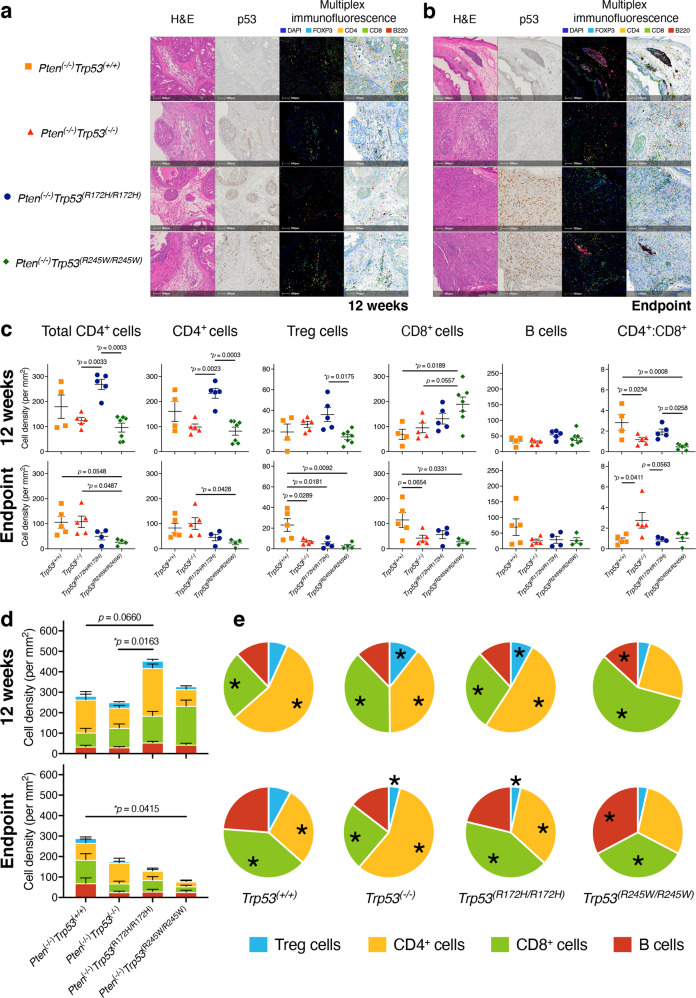
Immune cell profiles of mouse prostate cancers correlated with isogenic alterations of *Trp53*, in the absence of *Pten*. Whole Formalin-Fixed Paraffin-Embedded (FFPE) prostate sections were stained by OPAL multiplex immunohistochemistry (mIHC) immune panel for FOXP3 (cyan), CD4 (yellow), CD8 (green), B220 (red) and DAPI (blue). Representative images showing H&E, p53 immunohistochemistry (IHC), mIHC, and mIHC-absorption of AP lobes of: **a** 12-weeks old mice; and **b** at ethical endpoint. Cell segmentation and phenotyping enabled quantitation expressed as density of total cells for different immune cell subsets relative to overall tissue size (cells/mm^2^). **c** Comparison between genotypes of immune cell densities calculated from total cell counts of identified immune subsets in AP lobes of 12-weeks old mice and in AP lobes at the ethical endpoint. **d** Cumulative bar plots of mean immune cell densities for AP lobes at 12-weeks of age and at ethical endpoint, respectively. *p*-values were calculated using ANOVA and Tukey’s tests. Error bars indicate mean ± SEM. **e** Comparison of the relative proportion of immune cells between AP lobes of 12-weeks old mice and AP lobes at the ethical endpoint. *p*-values were calculated using a two-tailed student’s *t*-test. Error bars indicate mean ± SEM. *Pten*^*(−/−)*^*Trp53*^*(+/+)*^: 12-weeks old mice *n* = 4 and endpoint mice *n* = 5; *Pten*^*(−/−)*^*Trp53*^*(−/−)*^: 12-weeks old mice *n* = 5 and endpoint mice *n* = 5; *Pten*^*(−/−)*^*Trp53*^*(R172H/R172H)*^: 12-weeks old mice *n* = 5 and endpoint mice *n* = 4; *Pten*^*(−/−)*^*Trp53*^*(R245W/R245W)*^: 12-weeks old mice *n* = 7 and endpoint mice *n* = 4.

As the status of *TP53* impacts the immune microenvironment (reviewed [15]), and the type and extent of immune cell infiltration in PC is clinically relevant [37]; we measured these parameters in the PC model null for *Pten* and homozygous for *TP53* isogenic alterations. With the *Pten* null status universal in the following comparisons, the findings are described on the basis of *Trp53* status alone. We adopted multiplex immunofluorescence labelling of CD4^+^ T cells, Tregs (T regulatory cells CD4^+^, Foxp3^+^), CD8^+^ T cells, and B cells (B220^+^), at 12 weeks and ethical endpoints in the mouse APs tumours (Fig. 4a, b, respectively). At 12 weeks of age (Fig. 4c), total CD4^+^ cells were at highest density in *Trp53*^*(R172H/R172H)*^ tumours, reflecting the high levels of both the consitutent CD4^+^ cells and Tregs, relative to the other genotypes. They were also the most abundant cell population in this genotype (Fig. 4d). CD8^+^ cells dominated *Trp53*^*(R245W/R245W)*^ tumours, with levels significantly higher than the *Trp53*^(+/+)^ controls and *Trp53*^*(−/−)*^ tumours at 12 weeks (Fig. 4c, d). At endpoint, *Trp53*^(+/+)^ tumours had the highest immune cell infiltration (comprising all the four immune cell populations CD4^+^, CD8^+^, B cells and Tregs), followed by *Trp53*^*(−/−)*^, *Trp53*^*(R172H/R172H)*^ and *Trp53*^*(R245W/R245W)*^ cohorts, respectively (Fig. 4c, d).

Overall immune cell population density of *Trp53*^(+/+)^ tumours was similar between 12 weeks and endpoint (Fig. 4d), although with time, there was an increase in CD8^+^ cells at the expense of CD4^+^ cells (Fig. 4e). In contrast, overall immune cell populations dropped markedly by their endpoints among tumours with *Trp53* alterations (Fig. 4d). This was accompanied by changes in the relative proportions of immune cell populations, across time (Fig. 4e). Explicitly, the proportion of CD8^+^ cells increased over time for *Trp53*^*(R172H/R172H)*^ tumours at the expense of CD4^+^ cells; for *Trp53*^*(R245W/R245W)*^, the relative proportion of B cells increased at the expense of the CD8^+^ cell populations; for *Trp53*^*(−/−*)^ tumours while the denisty of CD4^+^ cell populations was similar between timepoints, the proportion increased at endpoint, due to concomitant lowering of the relative fraction of CD8^+^ cells.

**Fig. 5:**
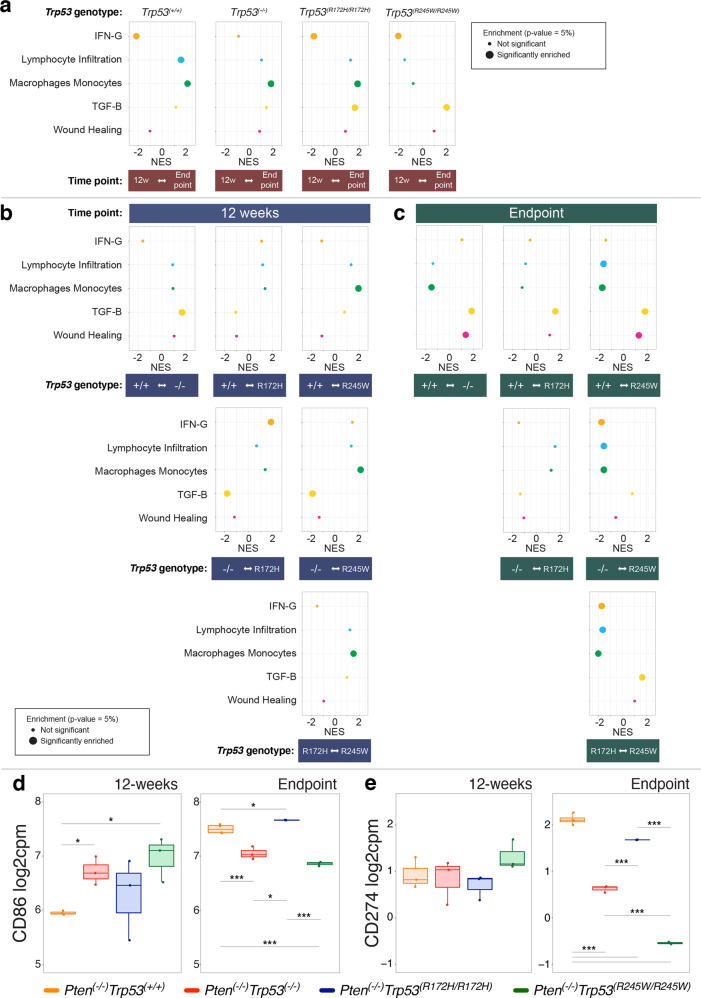
Immune gene expression signatures vary between prostate tumours in mice dependent on their isogenic *Trp53* alterations. Gene set enrichment analysis (GSEA) for relative immune signature expression among *Trp53* isogenic variants in the mouse PC tumours at 12 weeks and endpoints. Comparisons between the isogenic cohorts are shown as normalised enrichment scores (NES) for AP tumours at: **a** 12 weeks relative to endpoint; **b** 12 weeks; and **c** endpoint. Significance was designated as Benjamini–Hochberg adjusted *p*-value < 0.05 and demarked by a proportionately large dot. Comparison of: **d** *CD86*; and **e** *CD274* expression in AP tumours at 12 weeks and endpoint. Two sided *t*-tests were used to compare between groups, with significance levels indicated as: *p < = 0.05, **p ≤ 0.01, ***p ≤ 0.001. One-way Anova was used to compare all groups with *p*-values indicated. *Pten*^*(−/−)*^*Trp53*^*(+/+)*^: 12-weeks old mice *n* = 3 and endpoint mice *n* *=* 4; *Pten*^*(−/−)*^*Trp53*^*(−/−)*^: 12-weeks old mice *n* = 3 and endpoint mice *n* = 3; *Pten*^*(−/−)*^*Trp53*^*(R172H/R172H)*^: 12-weeks old mice *n* = 3 and endpoint mice *n* = 3; *Pten*^*(−/−)*^*Trp53*^*(R245W/R245W)*^: 12-weeks old mice *n* = 3 and endpoint mice *n* = 3.

Gene expression profiles provide a complementary approach to measuring immune responses, as we have previously performed [38, 39]. Studying immune signature pathways across genotypes (Fig. 5), allowed us to distinguish how p53 LOF impacts when measured relative to control mice; but also, GOF properties for the specific mutants, when evaluated relative to p53 null mice. A comparison across time revealed a decrease in *IFN-Gamma* in all genotypes at endpoint, implying that this is a *Pten*^(*−/−)*^ feature (Fig. 5a). *TGF-B* enrichment was evident in *Trp53*^(*−/−*)^ tumours at 12 weeks (Fig. 5b) and in all *Trp53* isogenic tumours at endpoint (Fig. 5c). The most striking finding at the 12 week time point was the significant macrophage/monocyte profile dominating *Trp53*^*(R245W/R245W)*^ tumours (Fig. 5b). In the context of cancer, the type of macrophage populations are influential.

Toward defining the particular macrophage populations dominating *Trp53*^*(R245W/R245W)*^ tumours, we compared individual immunomodulating gene profiles (as described in [38]), across time points and between genotypes (Supplementary Fig. 4). Pertinently, the 12 week (Supplementary Fig. 4b) analyses revealed elevated levels of a number of individual genes among these tumours that are relevant to a tumour-associated macrophage gene expression signature (sometimes also referred as to M2-like; anti-inflammatory gene signature linked to tumour progression). Notably, relative to the other genotypes at the same 12 week time point, in *Trp53*^*(R245W/R245W)*^ tumours, RNA levels were elevated for: (1) *TGFB1*, whose product TGF-Beta is secreted by tumour associated M2-type macrophages, as noted in gliomas, where it promotes stemness and migration [40]. Consistently, TGF-Beta beta drives advanced PC through the promotion of EMT and metastasis (reviewed in Ref [41]). (2) Intracellular Adhesion Molecule 1 (ICAM-1) has been correlated with M2-macrophage polarization, as identified in lung cancer [42]. In addition, migration and invasion of mutant p53 PC cells is promoted by their exosomal packaging of ICAM-1 [43]. This data suggests that at 12 weeks, *Trp53*^*(R245W/R245W)*^ tumours exhibit a novel GOF linked to the accumulation of tumour associated macrophage-monocyte populations.

In addition, at 12 weeks in *Trp53*^*(R172H/R172H)*^ and *Trp53*^*(R245W/R245W)*^ tumours, the Tumour Inflammation Signature analyses (described in [38]; Supplementary Fig. 5a–c) identified elevated expression of *CCL5* (C-C chemokine ligand 5; Supplementary Fig. 5b), which is produced by tumour associated macrophages. CCL5 drives EMT, invasion and migration in PC, plus the self-renewal of PC stem cells [44]; with antagonists of its receptor CCR5 in development for therapy (reviewed [45]). Of note, elevated *CCR5* expression has been closely linked to EMT in human PC, associated with high-grade progressive disease (demarked by N-Cadherin upregulation and E-cadherin down regulation [46]).

An additional and notable GOF in *Trp53*^*(R172H/R172H)*^ and *Trp53*^*(R245W/R245W)*^ tumours, is the elevation of *PSMB10* at 12 weeks (Supplementary Fig. 5b). PSMB10 is a 20 S core subunit of the 26 S proteasome. Expression of multiple immunoproteasome subunits, including *PSMB10* have been linked to mutant p53 in breast cancer [47]. *PSMB10* overexpression has also been associated with angiotensin II and inflammation in numerous cancers although not previously reported for PC, as far as our literature search has extended [48].

In contrast, at endpoint, individual gene analyses identified reduction in expression of *TGFB1*, accompanied by reduced levels of *IFNGR1* and elevation of mRNA levels of ICAM-1. This was observed for all the *Trp53* isogenic cohorts relative to the wt *Trp53* controls (Supplementary Fig. 4c). Notably, loss of *IFNGR1* promotes tumour cell proliferation in melanomas and diminishes IFN-Gamma induced apoptosis [49]. We point out these co-occurent gene alterations above others, as they were the common endpoint responses when *Trp53* was deleted or mutated, consistent with the loss of wt p53 tumour suppressor function.

Immune signatures of *Trp53*^*(−/−*)^ and *Trp53*^*(R172H/R172H)*^ tumours were not significantly distinguishable from each other at endpoint (Fig. 5c). On the other hand for *Trp53*^*(R245W/R245W)*^ tumours, immune signatures for IFN-Gamma, lymphocyte infiltration and macrophages/monocytes were lower. In brief, the expression profiles of the genes of these pathways trended in a manner indicative of a *Trp53* mutant-specific GOF. In contrast, the TGF-Beta pathway signature was significantly higher in *Trp53*^*(R245W/R245W)*^ relative to *Trp53*^*(R172H/R172H)*^ tumours (Fig. 5b). A number of other genes differed in expression between the mutants at endpoint; however, their lack of significant expression levels relative to the controls, caused this to be less easily interpreted and we chose not to focus on these in this study (Supplementary Fig. 4c).

Immune checkpoint blockade has not proven advantageous in PC and robust markers of efficacy are lacking [50]. Blocking ligand engagement of checkpoint receptors PD-1 and CTLA-4 are the key treatment strategies in trials for reinvigorating exhausted immune responses. Levels of these ligands are emerging among the tool kit of biomarkers predictive of treatment efficacy, at least in colon tumours (reviewed [51]), which prompted us to question correlations in PC. To investigate the possibility that the ligands of these receptors accumulate at different levels and stages in the *Trp53* isogenic tumours, we quantified expression in tumours at 12 weeks and endpoint (Fig. 5d, e). The CTLA-4 ligand, CD86 (*CD86* mRNA), was significantly elevated in tumours from *Trp53*^*(R245W/R245W)*^ and *Trp53*^*(−/−*)^ aged 12 weeks, relative to the wt *Trp53* controls (Fig. 5d). For the *Trp53*^*(R172H/R172H)*^ mutant, although *CD86* levels were generally higher at 12 weeks, they were significant only at endpoint, by which stage those of *Trp53*^*(R245W*/R245W)^ and *Trp53*^*(−/−*)^ were lower than the controls. These data suggest a distinct temporal influence of these specific p53 mutants and predict individual, time-dependent therapeutic opportunities. It is notable that low levels of *IFNGR1* (encoding IFN-Gamma) (Supplementary Fig. 4c; as measured in all the *Trp53* isogenic tumours relative to the controls at endpoint), has been associated with poor response to anti-CTLA-4 therapy (ipilimumab) in melanomas [49]. PD-L1 (encoded by *CD274* mRNA) had a selective trend to elevation in the *Trp53*^*(R245W/R245W)*^ tumours at 12 weeks, although it did not reach significance. At endpoint, *CD274* levels were below those of the control for all samples lacking wt p53 (Fig. 5e).

### PC *Trp53* isogenic mice uncover alterations in cancer pathways and expose candidate drug targets

**Fig. 6:**
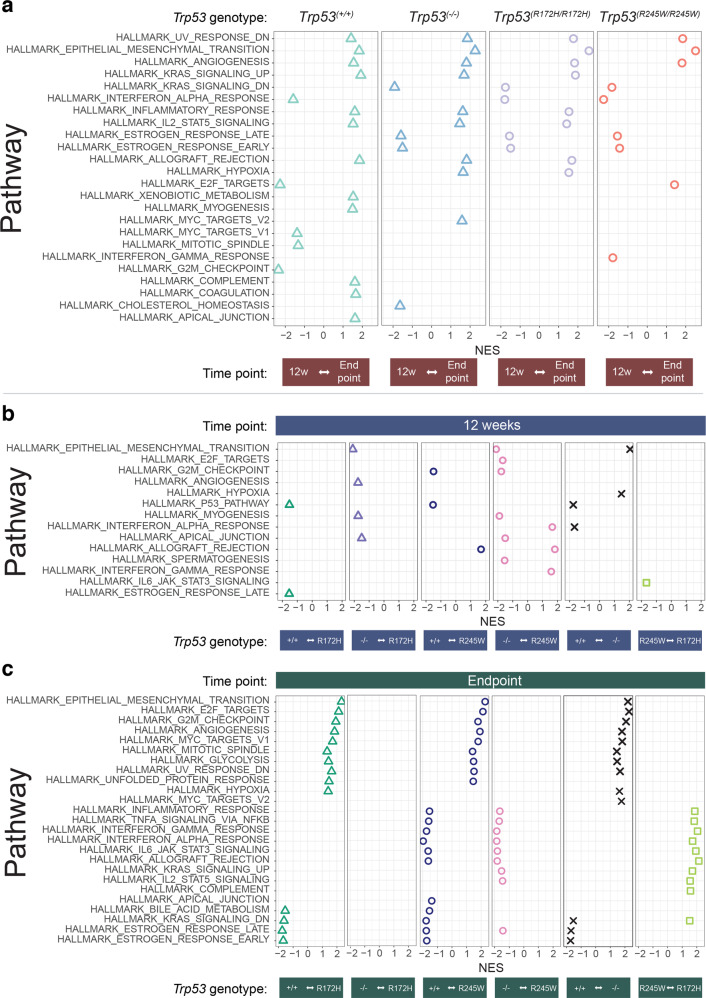
Hallmark signature gene set analyses revealed temporally distinct pathway enrichment between PCs of the *Trp53* isogenic variants. Gene set enrichment analyses (GSEA) for HALLMARK pathways at: **a** 12 weeks relative to endpoint; **b** 12 weeks; and **c** endpoint. Comparisons are plotted as relative normalised enrichment scores (NES). Only pathways with Benjamini–Hochberg adjusted *p*-values < 0.05 are shown. *Pten*^*(−/−)*^*Trp53*^*(+/+):*^ 12-weeks old mice *n* = 3 and endpoint mice *n* = 4; *Pten*^*(−/−*)^*Trp53*^*(−/−):*^ 12-weeks old mice *n* = 3 and endpoint mice *n* = 3; *Pten*^*(−/−)*^*Trp53*^*(R172H/R172H)*^: 12-weeks old mice *n* = 3 and endpoint mice *n* = 3; *Pten*^*(−/−)*^*Trp53*^*(R245W/R245W)*^: 12-weeks old mice *n* = 3 and endpoint mice *n* = 3.

Gene Set Enrichment Analysis (GSEA) of Hallmark cancer gene sets [52, 53], among all tumours, consistently identified that a *Pten*^*(−/−*)^ background produced endpoint signatures of downregulated UV response, elevated EMT, angiogenesis, inflammatory response and IL-2-STAT5 signalling, and overall higher KRAS signalling, in contrast to their 12 week counterparts (Fig. 6a). The addition of genetic alterations of *Trp53* resulted in reduced oestrogen responses at endpoint (Fig. 6a).

Changes to normal p53 function in tumours were already evident at 12 weeks, as indicated by significantly lower expression of p53 Pathway genes for *Trp53*^*(R245W/R245W)*^, *Trp53*^(*R172H/R172H*)^ and *Trp53*^*(*−/−)^, relative to the *Trp53*^*(+/+)*^ controls (Fig. 6b). No other pathways were universally perturbed between all the *Trp53* isogenic variants at this point. Expression of the EMT signature in the *Trp53*^*(*−/−)^ tumours was pronounced relative to the isogenic mutants and the *Trp53*^*(+/+)*^ controls at 12 weeks (Fig. 6b), and then became elevated in all *Trp53* variants at endpoint (Fig. 6c).

At endpoint, alteration of *Trp53* caused additional changes to many vital cancer pathways, notably: E2F Targets, G2M Checkpoint, Angiogenesis, MYC targets v1, Mitotic Spindle, Glycolysis and Down regulationion of UV Response. Validating the models, the Unfolded Protein Response was elevated only for the *Trp53* mutants (Fig. 6c). Genotype-specific changes of immune signatures were identified in endpoint tumours and we focus on these as consistent with the theme of the study. Notably, *Trp53*^*(R245W/R245W)*^ tumours exhibited lower levels of a number of immune responses specifically relevant to cancers, compared with the *Trp53*^*(+/+)*^, *Trp53*^*(−/−)*^ or the *Trp53*^(*R172H/R172H*)^ counterparts; examples include: inflammatory response; TNF-Alpha signalling via NFK-Beta; IFN-Gamma response; IFN-Alpha response; and IL6-JAK-STAT3 signalling (Fig. 6c).

Also profoundly downregulated in the *Trp53* altered tumours, relative to the *TP53*^*(+/+)*^ controls at endpoint, were the Oestrogen Responses (Fig. 6c and also relevant to Fig. 6a). Pertinently, p53-oestrogen links have been established, with oestrogen receptor-beta performing a protective role (as reviewed [54]). Furthermore, normally the oestrogen receptor-beta normally represses androgen receptor activity and loss of this control is a major driver of PC proliferation, consistent with aggressive disease [55].

## Discussion

We have uncovered a remarkable influence of age, on the incidence of individual mutations of the tumour suppressor gene *TP53* in human PC. p53 mutations at amino acid residues R248 and R175 were the most prevalent in PC in men ≤50 years, coincident with metastatis (Fig. 1a). Critically, metastatic PC is linked to poor outcomes (reviewed [13]) and this is particularly pertinent to younger men with high grade and stage disease (as reviewed [56]). The rates of PC in younger aged US men is increasing, without adequate explanation [57]. Our PC mouse models provide a clinically relevant context for exploring human PC at younger age.

In greater detail, our PC mouse models align with the PC data in men ≤50 years, where disease frequency and metastases were most strongly associated with mutation of *TP53*^*(R248)*^ over *TP53*^*(R175)*^ (Fig. 1). Pertinently, these findings are consistent with the relative influence of these mutants in a breast cancer model developed in the Lozano lab [58]. It is notable that a comprehensive pan-cancer study identified R273, R248 and R175 as the most consistently altered amino acids in p53 from both the Sanger data set and also TCGA [24]. In PC specifically, we found that the prevalence of p53 mutation R273 increased in prominence after age 50 and then dominated older age PC. This is relevant to the recently published PC model from the Knudsen lab, based on cBioportal PC patient data [59]. Our studies reveal the distinct age contextual relevance of each of these PC mouse models.

Our new PC models provide a unique, clinically relevant tool for characterising the genetic-molecular changes occurring between the selected *Trp53* isogenic tumours, across the time of disease onset and progression. These isogenic, orthotopically-engineered temporal changes, from wt to altered *Trp53*, in an immune-competent context, offer a remarkable simulation model for garnering insight into the events occurring in malignant, treatment resistant, lethal human PC.

Human prostate tumour microenvironments are comprised of both immune and non-immune cells (e.g. stroma) that undergo dynamic transcriptional reprograming under the influence of the associated PC cells ([10]; reviewed in [60]). This is of particular relevance to new, in vivo cancer studies that have unveiled critical roles of tumour suppressors in driving anticancer immunity. Notably, *PTEN* loss has been linked to an immunosuppressive environment in PC [16], including a weakening of the interferon network [17]. Consistently our data correlated *Pten*^*(−/−)*^ *to* reduction in the Interferon-gamma Hallmark in all genotypes at endpoint (Fig. 5a). Concommitantly, *Pten* loss led to an increased rate of proliferation (Supplementary Fig. 2a, Table f). Pertinently, deleting tumour suppressors was found to be central to the capacity of cancer cells to proliferate, following transplantation into intact immune mice models (e.g. [61]), and while this is relevant to Pten, the situation is more complex for p53.

In contrast to *PTEN* deletion occuring commonly in human PC, *TP53* has been identified to undergo deletion and mutation (reviewed [13]). *TP53* mutations are most commonly missense, which are a mutation class frequently associated with the acquisition of oncogenic functions [62]. As we reviewed [15], p53 is a vital regulator of immunity, with major perturbations associated with *TP53* mutations.

Of critical relevance, immunoregulatory capacities of tumour suppressors have been severely overlooked in traditional in vitro, 2D analyses of mutant *TP53* cell lines. One example is that the Achilles project failed to identify mutant p53 GOFs in high-grade serous ovarian cancer, despite its near universal prevalence [63] and established link to poor patient survival [64]. A second example is that whole genome CRISPR KO screens did not identify *TP53* mutant GOFs in hematopoietic cells [65]. It is also worth noting that while in vivo screening of these cells failed to identify GOFs, subsequent studies identified that the mouse strain adopted for this work displays certain immune incompetencies (e.g. altered lymphocyte profiles [66] and also possibly faulty natural killer cells [67]). Importantly, mutant p53 was separately demonstrated to increase risk of haematological malignancies in involving epigenetic mechanisms [68]. These examples emphasize the need for appropriate pre-clinical models to explore the pervasive genetic selection for *TP53* mutation in cancers. Our new PC mice model simulates orthotopic tumour growth that is limited to the prostate epithelium, arising in an immune-competent microenvironment, enabling the exploration of tumour suppressor activities and the impact of genetic alterations that are relevant to human disease.

We focused on the impact of *Trp53* status on tumour development in an immunologically intact context. The novelty of this approach is that traditional xenograft models or others with whole body alterations of *Trp53*, have been incapable of competently simulating these features. Importantly, we identified a time-dependent disease manifestation in a *Trp53* isogenic-dependent manner. Mutant p53 protein stabilisation was most pronouced in *Trp53*^*(R245W/R245W)*^ tumours and critically this corresponded with the most aggressive manifestation of mutant p53 GOF. On a background of *Pten*^*(−/−)*^ in response to *Trp53*^*(R245W/R245W)*^ mutation, tumour proliferative growth was accelerated relative to the other isogenic strains from ~21 weeks onwards (Supplementary Fig. 2a, Table f) and ultimately corresponded with the heaviest tumours at endpoint (Fig. 2b). The more aggressive nature of mutant *Trp53*^*(24*5)^ over mutant *Trp53*^*(172)*^ provides excellent correspondence to the human counterparts.

The validity of these models was further supported by the uniform low expression of the P53 pathway Hallmark across the tumours with altered *Trp53*, as emerged at 12 weeks (Fig. 6b). It is notable that at this early time point there were no other uniform changes across the *Trp53* isogenic tumours. This contrasts with endpoint, where multiple signatures were affected (Fig. 6c).

Differences in Hallmark signature expression among the *Trp53* isogenic mice at 12 weeks, is consistent with temporal variation in rates of disease manifestation. *Trp53*^*(R245W/R245W)*^ tumours at 12 weeks, exhibited exceptional: tumour weight increase (Fig. 2a); infiltration of CD8^+^ T cells (Fig. 4c–e); and the gene signature of M2-like macrophages (Fig. 5). Importantly, these features of *Trp53*^*(R245W/R245W)*^, are relevant to the characteristics of very high risk PC in humans [69]. At endpoint, p53 levels were most highly accumulated in *Trp53*^*(R245W/R245W)*^ tumours relative to the counterpart isogenic strains (Fig. 3b) and this corresponded with lowest immune cell density (Fig. 4d) and greatest tumour weight (Fig. 2b). On the other hand, the slightly less proliferative *Trp53*^(*R172H/R172H*)^ tumours (Supplementary Fig. 2a, Table f) provoked a greater immune response at 12 weeks, which was dominated by CD4^+^ T cells (Fig. 4c–e). By endpoint this immune reaction was profoundly subdued (Fig. 4c, d). *Trp53*^*(−/−)*^ tumours by contrast, had a slower proliferation rate (Supplementary Fig. 2a, Table f) and at 12 weeks displayed immune responses closer to the wt p53 controls (Fig. 4c, d), compared with the isogenic mutant tumours. Higher overall immune cell density levels in *Trp53*^*(−/−*)^ tumours were maintained at endpoint relative to the mutant counterparts (Fig. 4c, d). These isogenic comparisons offer a robust system for differentiating novel oncogenic activities imposed in a *Trp53* mutant-specific manner, as compared to its loss (in a *Pten* loss context).

An intriguing finding is that at endpoint, tumour-associated lymph nodes had increased size, weight and cellularity in correlation with the accumulation of p53 levels in the corresponding primary tumours, and generally also with these tumour weights (Supplementary Fig. 3c, relative to Fig. 3 and Fig. 2b). Importantly, while aggressive tumours such as *Trp53*^*(R245W/R245W)*^ provoked intense immune cell accumulation in their associated nodes where *Trp53* was not mutated, immune cells were very poorly penetrant into the endpoint tumours themselves, where mutant p53 accumulated. This is consistent with mutant p53 in the tumour cells actively suppressing host immunity in the immediate vicinity. In keeping with this, our gene expression analyses comparing the different tumour genotypes, strongly suggested that core immunological pathways were sensitive to the status of p53 and in some cases were influenced by different mutations (Fig. 6c). Establishing a temporal scale of immunological changes that are dictated by individual *Trp53* mutations, holds potential for opening a deeper level of personalising treatments for aggressive human PC. An example of this is the elevation of the IL6-JAK-STAT3 signalling Hallmark in *Trp53*^*(R245W/R245W*)^ tumours at 12 weeks relative to *Trp53*^*(R172H/R172H)*^ (Fig. 6b), followed by an endpoint decline (Fig. 6c). This pathway drives chronic inflammation and immunosuppression, and is a good candidate for further study and/or clinical application (i.e. [70]). Together these findings indicate strong immunosuppressive GOFs, with the timing of influence and the levels of response correlating with the nature of the mutation. In turn, this hints at the exciting possibility of recruiting a strong immune response, if such local inhibitory barriers within the tumour can be overcome.

Analysis of other Hallmark signatures revealed significant alterations particularly at endpoint related to cell cycle, metabolism and transformation associated with invasive capacity (Fig. 6c). Of relevance, comprehensive TCGA Pan cancer analyses exposed that mutation of *TP53* correlated with significantly upregulated expression of genes that drive cell-division. Notably, among these were promoters of cell cycle, mostly those involved in regulating the G2/M checkpoint; and E2F target genes (where E2F is an important S phase/G2 promoting transcription factor) that is repressed by wt p53 [24]. Our data align with these findings and add the observation of increased glycolysis, as consistent with the rapid energy generating Warburg phenomenon, being promoted by *Trp53* mutation [71], in contrast to its normal suppression by wt p53 [72] (and reviewed [73, 74]).

Acquisition of invasive capacity was first evident in *Trp53*^*(−/−)*^ tumours at 12 weeks, in contrast to the *Trp53* mutants, which only displayed EMT at endpoint, both through protein staining (vimentin positivity Fig. 3) and gene expression (Fig. 6c). This clearly demonstrates that wt p53 robustly suppresses EMT in PC. EMT is a critical step in the malignant progression of invasion and metastasis (reviewed [75]), as is the acquisition of new vasculature consistent with angiogenesis (Fig. 6c). These age and genotype dependencies for EMT onset (Fig. 6b, c), add further to the prediction that treatment-timing is likely to be a vital determinant of its efficacy.

We have shown that *TP53* mutation-type is a clinically relevant consideration that warrants further investigation in the context of PC precision medicine, particularly in the era of immune therapies. Our data predicts that early intervention with CTLA-4 blockade therapy is valuable to explore in human PC with altered *TP53*. The relevance of testing early intervention for CTLA-4 targetting is emphasized by the absence of benefit among mCRPC patients [50]. In contrast, anti-PD-L1 checkpoint blockade therapy for PC patients is not predicted to be significantly beneficial where tumours have altered *TP53*, and particularly not for patients with advanced disease stage, where PD-L1 levels are predicted to be universally low. These findings for PD-L1 align with the poor response of mCRPC patients to its antibody targeting as a monotherapy [50]. Our findings argue for *TP53* sequencing of PC patient tumours in order to better understand disease development and in turn to rationally personalise treatment, including the potential for new immunotherapies.

## Materials and methods

### Ethics statement

Animals were cared for and treated in accord with the Australian Code of Animal Care and the Use of Animals for Scientific Purpose. Experiments involving mice were conducted with approval from the Peter MacCallum Cancer Centre Animal Experimentation Ethics Committee (E589 & E625).

### Mice

A PC-specific mouse model was constructed using the Cre-loxP system to drive alterations of the two key tumour suppressors *Pten* and *Trp53*, on a C57BL/6 background (99.61% C57BL/6; N8). Introduction of Cre recombinase (Cre) engineered with a probascin (Pb) sensitive promoter [76] directed localised genetic alterations to the epithelium of the prostate gland. Pb and cre were each introduced in a heterozygous manner exclusively in male mice to avoid recombination of the loxP-flanked alleles in other tissues the than prostate epithelium [77].

Prostate-specific homozygous *Pten* deletion [78, 79] was the universal context for four *Trp53* isogenic mice genotypes. Wild-type (wt) *Trp53* was either knocked out [80, 81] or substituted using a sophisticated genetic construct that allows wt *Trp53* expression, until Cre exposure induces the switch for mutation expression. These switched alleles have been defined in the mice as *Trp53*^*wm-R172H*^ and *Trp53*^*wm-R245*^ where wm indicates wt to mutant transition as previously described for a breast cancer model induced locally by viral Cre (where an arginine to histidine substitution at codon 172, results from a CGC to CAC nucleotide change; where an arginine to tryptophan change at codon 245, results from a CGC to TGG nucleotide change [58, 82]). For simplicity, the four *Trp53* genotypes are referred to as: *Trp53*^*(+/+)*^; *Trp53*^*(−/−)*^; *Trp53*^*(R172H)*^; *Trp53*^*(R245W)*^ (where the human equivalent of the mutant alleles are *TP53*^*(R175H)*^ and *TP53*^*(R248W)*^ respectively).

### Mouse maintenance and husbandry

Mice were housed at the Peter MacCallum Cancer Centre animal house in a pathogen-free environment, in a 12 h light/dark cycle at 23 °C ± 2 °C room temperature with a relative humidity of 50 ± 20%, and unrestricted access to food and water. Environmental enrichment was provided consistently across cages. Mice were monitored routinely and sacrificed ethically in compliance with the Australian Code of Animal Care and the Use of Animals for Scientific Purpose.

### DNA isolation and genotyping

Genotyping was performed on mouse tail DNA as previously described [83], using the listed primers (Table 1). PCR programmes for all the primers pairs were set at: Initialisation 94 °C for 5 min; Denaturation 94 °C for 1 min; Annealing 60 °C for 1 min; Extension 72 °C for 1 min; Final elongation 72 °C for 8 min; 35 cycles. PCR products were separated by 2% agarose electrophoresis (Catalogue No. BIO-41025, Bioline) and visualised using MIDORIGreen Advance (Catalogue No. MG04, NIPPON Genetics EUROPE) staining. Cre genotyping primers produce a single band if positive and no band if negative; thus, *IL2* was used as amplification-internal control as described before [84].

**Table 1:** Primers sequences for gene detection in genomic DNA.

Target	Sequence	Predicted amplicon size band
*Pb-Cre*	Forward 5'-TCT GCA CCT TGT CAG TGA GG-3' Reverse 5'-ATG TTT AGC TGG CCC AAA TG-3'	480bp
*IL2*	Forward 5'-CTA GGC CAC AGA ATT GAA AGA TCT -3' Reverse 5'-GTA GGT GGA AAT TCT AGC ATC ATC C-3'	325bp
*Pten*	Forward 5'-CTT CGG AGC ATG TCT GGC AAT GC-3' Reverse 5'-AAG GAA GAG GGT GGG GAT AC-3'	974bp for *Pten*^fl/fl^ ; 811bp for *Pten*^wt/wt^
*Trp53*	Forward 5'-AAG GGG TAT GAG GGA CAA GG-3' Reverse 5'-GAA GAC AGA AAA GGG GAG GG-3'	550bp for *Trp53*^fl/fl^ ; 430bp for *Trp53*^wt/wt^
*Trp53*^ΔwmR172H^	Forward 5'-ACCT GTA GCT CCA GCA CTG G-3' Reverse 5'-ACA AGC CGA GTA ACG ATC AGG-3'	420bp for *Trp53*^R172H/R172H^ and 340bp for *Trp53*^wt/wt^
*Trp53*^ΔwmR245W^	Forward 5'-ACC TTA TGA GCC ACC CGA -3' Reverse 5'- GGA AGA CAC AGG ATC CAG GT-3'	460bp for *Trp53*^R245W/R245W^ ; 420bp for *Trp53*^wt/wt^

Genotyping was performed on mouse tail DNA using the primers listed in Table 1. Please refer to DNA isolation and genotyping in Material and Methods for further details.

### Tissue collection and dissection

At designated time points (6, 12 weeks-old, or ethical endpoint, respectively) mice were weighed, euthanised by cervical dislocation. Abdominal swelling (due to primary tumour) was monitored weekly and humane end-point intervention was undertaken when the total prostate tumour volume per mouse reached ~1500 mm^3^ (as gauged by a length of typically ~30 mm). The genitourinary bloc (GU-bloc) consisting of the prostate lobes, seminal vesicles, bladder, proximal ductus deferens, and proximal urethra was dissected *en bloc* as described [85] and weighed (with an emptied bladder). For the collections at 6 and 12 weeks of age, the anterior prostate (AP) lobes were clearly differentiated, which allowed their unambiguous dissection, as previously described [86], then weighing. This was not possible for late time points, where disease had distorted the prostate lobe architecture. Tissues were photographed and immediately fixed in 10% neutral buffered formalin at room temperature. After 24 h, the fixed tissues were stored in 70% ethanol at 4 °C in advance of processing for paraffin-embedding, histology, immunohistochemistry and OPAL multiplex analysis.

### Histology and immunohistochemistry

Tissues were cut at a thickness of 4 μm, deparaffinised with xylene, rehydrated, stained with hematoxylin and eosin (H&E), or used for immunohistochemistry. p53, vimentin, cytokeratin 5 (CK5) and p63 immunohistochemistry (IHC) was performed on fixed and embedded tissues that were first processed for antigen unmasking by boiling in Dako EnVision FLEX High pH TRS (Agilent Technologies), pH 9.0, for 20 min at 100 °C, using a Dako PT Link (Agilent Technologies). All sections were incubated with Dako EnVision FLEX Peroxidase Blocking Solution (Agilent Technologies), at room temperature for 10 min followed by primary antibodies respectively. The antibodies were directed against p53 (Clone: FL393, Catalogue No. SC-6243, Santa Cruz Biotechnologies; at 1:100 dilution), Vimentin (Clone: SP20, Catalogue No. ab16700, Abcam; at 1:100), CK5 (Clone: 2C2, Catalogue No. MA517057, Thermo Fisher Scientific; at 1:500), and p63 (Clone: DAK-p63, Catalogue No. M731701-2, Agilent Technologies; at 1:50). Primary antibody incubation for p53 was performed overnight at 4 °C, while all other primary antibody incubations were done at room temperature for 30 min. Antibody binding for p53 and Vimentin was detected using secondary goat anti-rabbit IgG (H + L) (Thermo Fisher Scientific; at 1:100 dilution), while binding for CK5 and p63 was detected using Dako EnVision FLEX HRP (Agilent Technologies) at room temperature for 30 min. Visualisation was completed using Dako EnVision FLEX DAB + Chromogen and copper enhancer. Due to the cross-reaction between the EnVision FLEX polymer mouse/rabbit HRP, these negative controls were used to assess antibody specific staining verses that of the secondary. Both mouse and human control tissues were used along with negative control sections where the primary antibody was omitted. Staining signals were visualised using the VS120-L100-W (OLYMPUS) system.

A certified veterinary pathologist (co-author F.Muntz,) was blinded to the mouse genotypes as she performed histopathological assessments of the tissues stained with H&E. A histoscore for p53 staining quantification was calculated by adding the value of the intensity to the proportion of cells stained with a final maximum score of 7. Intensity was scored on a scale ranging from 0 to 3. The proportion of cells stained was on a scale of 1–4 (0: no stain detected; (1) <10% stained; (2) 10–50% stained; (3) 50–80% stained; (4) >80% [35]).

### OPAL multiplex staining

Tissues were cut 5 μm in thickness and mounted on Trajan Series 3 adhesive slides. Slides were stained using antibodies against B220 (Catalogue No. 550286, BD Pharmingen; at 1:2000 dilution), CD4 (Catalogue No. 14-9766-82, eBioscience; at 1:1500 dilution), CD8 (Catalogue No. 14-0808, eBioscience; at 1:1000 dilution) and FOXP3 (Catalogue No. 14-5773-80, eBioscience; at 1:150 dilution) as recently described [87]. Stained slides were imaged using the Vectra® 3.0 automated quantitative pathology imaging system 3.0.5 (Akoya Biosciences). Images were spectrally unmixed in inForm 2.4.1 (PerkinElmer) using the appropriate spectral library.

### Immune cell profiling and cell counting

High resolution-whole tissue scans were uploaded into HALO® Image Analysis Platform (Indica Labs) software for cell densitometry analysis. Whole tissue sections for all samples were spatially segmented within the software according to the different prostate lobes [88]. However, the dorsal-lateral prostate (DLP) lobes and the ventral prostate (VP) lobes were enlarged and, in all cases, fused to the anterior prostate (AP) lobes, making it challenging to differentiate the different prostate lobes accurately. Therefore, the tissue was segmented either as AP lobes or as VP + DLP lobes for cell densitometry analysis. Identification of the AP lobes was easy as the disease is mainly manifested in these lobes. Prostatic glands were excluded from the analysis due to the high autofluorescence.

Cell segmentation was performed based on DAPI counter-stain. Cells were counted within either the AP lobes or VP + DLP lobes according to four cell phenotypes: (i) DAPI^+^CD4^+^CD8^-^B220^-^ (also referred as CD4^+^ cells); (ii) DAPI^+^CD4^-^CD8^+^FOXP3^-^B220^-^ (also referred as CD8^+^ cells); (iii) DAPI^+^ CD4^+^CD8^-^FOXP3^+^B220^-^ (also referred as Tregs); and (iv) DAPI^+^CD4^-^CD8^-^FOXP3^-^B220^+^ (also referred as B cells). The number of identified cells in each tissue zone was normalized to the total surface area to generate cell densities.

### 3' RNA-seq gene expression profiling

RNA was purified from 50 μm Formalin-Fixed Paraffin-Embedded (FFPE) tissue sections (Catalogue No. 73504, Qiagen) according to the manufacturer’s instructions. RNA was then eluted from the columns in 20 μL of DEPC-Treated Water (Catalogue No. AM9906, Thermo Fisher Scientific). RNA quality was tested using a High Sensitivity RNA TapeStation assay (Agilent) with a DV200 cutoff of >50%. 150 ng of total RNA from each sample were used for genome-wide transcriptomic analyses as described previously [89, 90]. Reads were aligned to GRCm38 using hisat2 (2.0.4) in unpaired mode with -qc-filter 2. Gene level counts were quantified ht-seq (v0.9.1). Raw counts were converted to counts per million (CPM) and lowly expressed genes <5 CPM were removed. CPM counts were normalized using the RUVIII method (*k* = 10) within the ruv (v0.9.7.1) R package.

RNA-seq data can be found in https://www.ncbi.nlm.nih.gov/geo/query/acc.cgi?acc=GSE209532 (please refer to data availability statement).

### Gene mutation and gene ontology and pathway analyses

*TP53* mutations in prostate adenocarcinomas were analysed from the Catalogue of Somatic Mutations in Cancer (COSMIC) database [26]. Explicitly, COSMIC mutation data (release v95) from targeted and genome-wide screens was downloaded from https://cancer.sanger.ac.uk/cosmic/download on 3 December, 2021. Mutations were filtered to include missense mutations only. Samples were then grouped by tumour origin (primary and metastatic) and patient age and the number of samples with mutations in each codon was plotted using ggplot2 (v3.3.3).

Gene Set Enrichment Analyses (GSEA) [53] using the Molecular Signatures Database hallmark gene set collection (H: 50 gene sets) [52], was undertaken on differential gene expression analyses results for the four *Pten*^*(−/−*)^, *Trp53* isogenic cohorts: *Trp53*^*(*+/+)^, *Trp53*^*(−/−*)^, *Trp53*^*(R17H2/R172H*)^, *Trp53*^*(R245W/R245W*)^. Studies were undertaken at two distinct time points, 12 weeks and endpoint. In-depth immune pathway profiling was undertaken as previously reported by our group [38]. GSEA was applied using the R package fgsea (v1.12.0). The rank metric used was logFold Change*(1-Benjamini–Hochberg [BH] adjusted *p*-value). BH adjusted *p*-values from GSEA were derived. Significant gene sets were inferred, using a 0.05 significance level.

### Statistical analysis

Two-way comparisons were analysed using an unpaired and two-tailed Student’s *t*-test using Prism 9 (GraphPad). One-way analysis of variance (ANOVA) followed by a Tukey post-hoc comparison test was performed on comparisons of more than two conditions using Prism 9 (GraphPad). Data are presented, if not indicated elsewhere, as mean ± standard error of the mean (SEM). Statistical significance is indicated in all figures. False discovery rates were assessed according to Benjamini–Hochberg with significance defined as an adjusted *p*-value < 0.05 as previously reported [38].

### Reporting summary

Further information on research design is available in the Nature Research Reporting Summary linked to this article.

## Supplementary information

Supplementary File

Supplementary Table S2f

Reporting summary checklist

Figure S1

Figure S2

Figure S3

Figure S4

Figure S5

## Data Availability

The bulk RNA-seq data has been deposited in GEO-NCBI (a public functional genomics data repository) under the accession code GSE209532.
