# Supplementary material for: Modelling aggressive prostate cancers of young men in immune-competent mice, driven by isogenic Trp53 alterations and Pten loss
Source: Cell Death Dis. 2022 Sep 8;13(9):777. doi: 10.1038/s41419-022-05211-y (PMC9465983; doi:10.1038/s41419-022-05211-y)
Supplement: Supplementary file 1 — Supplementary File [file 41419_2022_5211_MOESM1_ESM.docx]

**Modelling aggressive prostate cancers of young men in immune-competent mice, driven by isogenic *Trp53* alterations and *Pten* loss**

**Supplementary Figure 1: Increased tumour growth is concentrated in anterior compartment of prostate.** (a) AP lobes were dissected from the rest of the lobes (Prostate minus AP: Prostate-AP). Prostate-AP weights normalised to the body weight of 6 weeks-old and 12 weeks-old mice with different genotypes. Matrix showing p-values (blue indicates p-values >0.05) resulting from of statistical comparison of genotypes using ANOVA and Tukey’s tests in 6-week old (upper panel) and 12-week old (lower panel) mice. Error bars indicate mean ± SEM (n= 4-7 mice). (b) Prostates were harvested at ethical endpoint from the mouse carcass and dissected en bloc. Representative photographs of the prostates at the ethical endpoint of mice with indicated genotype. Bladder (Bl), seminal vesicles (SV) and anterior prostate (AP) lobes are denoted for each prostate.

**Supplementary Figure 2.** **Combined *Pten* loss and *Trp53* mutation impacts growth rate of prostate tumours**. (a-e) Growth rate for the indicated genotypes depicted as prostate weight (normalised to bodyweight) as a function of time. **Table f** summarises PC tumour growth kinetics where K is the rate constant; Tau is the time constant, reciprocal of K; and doubling-time was calculated as ln(2)/K. The goodness of fit of the exponential curves was determined using both a robust sum of squares and a robust standard deviation of the residuals (RSDR). n is shown in Table f for each genotype.

**Supplementary Figure 3**. **Enlargement of Peyer’s patches and medial iliac lymph nodes in transgenic mice**.

(a) and (b) are representative images of a gross dissection at the ethical endpoint of an adult male mouse in a supine position. Peyer’s patches were found to be enlarged in all transgenic mice. Besides the Peyer's patches, (b) medial iliac lymph nodes (mi-LNs) were significantly enlarged. mi-LNs were examined through (c) hematoxylin and eosin (H&E) staining; however, histopathological examination of excised LNs showed no metastatic foci (as shown in the zoomed-in images). (d) The area of whole H&E mi-LNs sections was determined from microscopy images and plotted for each genotype. Matrix showing p-values result of an ANOVA and Tukey’s test; blue indicates p-value >0.05 whereas red indicates p-value <0.05. Error bars indicate mean ± SEM. Pten*^(+/+)^*Trp53*^(+/+)^* n=2; Pten*^(-/-)^*Trp53*^(+/+)^* n=9; Pten*^(-/-)^*Trp53*^(-/-)^* n=7; Pten*^(-/-)^*Trp53*^(R172H/-)^* n=6; Pten*^(-/-)^*Trp53*^(R172H/R172H)^* n=7; Pten*^(-/-)^*Trp53*^(R245W/-)^* n=5; Pten*^(-/-)^*Trp53*^(R245W/R245W)^* n=11. (e) Representative hematoxylin and eosin (H&E) stained and p53 immunohistochemistry sections from bladders (Bl) of normal [Pten*^(+/+)^*Trp53*^(+/+)^*] and mutant [Pten*^(-/-)^*Trp53*^(R172H/R172H)^*] mice. Histopathological analyses revealed that some bladders of the transgenic mice displayed mt-p53 expressing cells with histological features of carcinoma in situ (CIS). Invasion of the muscle layer was not observed.

**Supplementary Figure 4. Gene Set Enrichment Analysis (GSEA) for Immunomodulatory genes exposes temporal profile differences among the isogenic *Trp53* variants in PC tumours.** GSEA plots comparing differentially expressed immunomodulatory genes in anterior prostate (AP) tumours at: (a) 12 weeks AP relative to endpoint AP tumours; (b) 12 weeks AP and (c) at endpoint. Significantly enriched immune signatures (Benjamini–Hochberg adjusted p-value <0.05) are denoted by larger dot size. Pten*^(-/-)^*Trp53*^(+/+)^*: 12-weeks old mice n=3 and endpoint mice n=4; Pten*^(-/-)^*Trp53*^(-/-)^*: 12-weeks old mice n=3 and endpoint mice n=3; Pten*^(-/-)^*Trp53*^(R172H/R172H)^*: 12-weeks old mice n=3 and endpoint mice n=3; Pten*^(-/-)^*Trp53*^(R245W/R245W)^*: 12-weeks old mice n=3 and endpoint mice n=3.

**Supplementary Figure 5. Expression of Tumour Inflammation-Associated genes in PC tumours differ between the isotype variants of *Trp53.*** Gene Set Enrichment Analysis (GSEA) plots show logFC of Tumour Inflammation-Associated genes based on differential gene expression analyses in anterior prostate (AP) tumours at: (a) 12 weeks AP relative to endpoint AP tumours; (b) 12 weeks AP and (c) at endpoint. Significantly enriched genes (Benjamini–Hochberg adjusted p-value <0.05) are denoted by larger dot size. Pten*^(-/-)^*Trp53*^(+/+)^*: 12-weeks old mice n=3 and endpoint mice n=4; Pten*^(-/-)^*Trp53*^(-/-)^*: 12-weeks old mice n=3 and endpoint mice n=3; Pten*^(-/-)^*Trp53*^(R172H/R172H)^*: 12-weeks old mice n=3 and endpoint mice n=3; Pten*^(-/-)^*Trp53*^(R245W/R245W)^*: 12-weeks old mice n=3 and endpoint mice n=3.
