# Supplementary material for: Modelling aggressive prostate cancers of young men in immune-competent mice, driven by isogenic Trp53 alterations and Pten loss
Source: Cell Death Dis. 2022 Sep 8;13(9):777. doi: 10.1038/s41419-022-05211-y (PMC9465983; doi:10.1038/s41419-022-05211-y)
Supplement: Supplementary file 2 — Supplementary Table S2f [file 41419_2022_5211_MOESM2_ESM.pptx]

## Slide 1
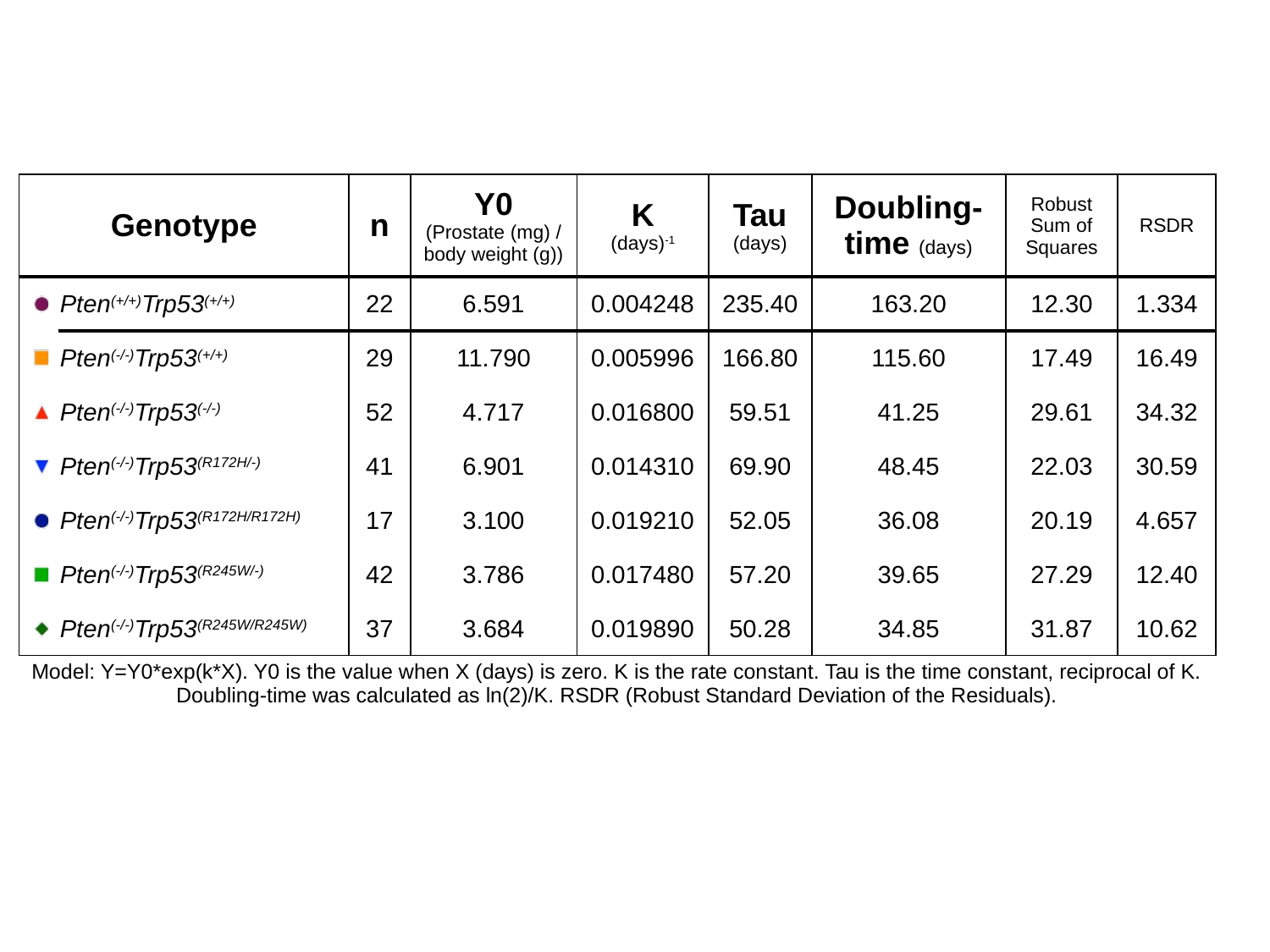

| Genotype | Genotype | n | Y0 (Prostate (mg) / body weight (g)) | K (days)-1 | Tau (days) | Doubling-time (days) | Robust Sum of Squares | RSDR |
| --- | --- | --- | --- | --- | --- | --- | --- | --- |
| | Pten(+/+)Trp53(+/+) | 22 | 6.591 | 0.004248 | 235.40 | 163.20 | 12.30 | 1.334 |
| | Pten(-/-)Trp53(+/+) | 29 | 11.790 | 0.005996 | 166.80 | 115.60 | 17.49 | 16.49 |
| | Pten(-/-)Trp53(-/-) | 52 | 4.717 | 0.016800 | 59.51 | 41.25 | 29.61 | 34.32 |
| | Pten(-/-)Trp53(R172H/-) | 41 | 6.901 | 0.014310 | 69.90 | 48.45 | 22.03 | 30.59 |
| | Pten(-/-)Trp53(R172H/R172H) | 17 | 3.100 | 0.019210 | 52.05 | 36.08 | 20.19 | 4.657 |
| | Pten(-/-)Trp53(R245W/-) | 42 | 3.786 | 0.017480 | 57.20 | 39.65 | 27.29 | 12.40 |
| | Pten(-/-)Trp53(R245W/R245W) | 37 | 3.684 | 0.019890 | 50.28 | 34.85 | 31.87 | 10.62 |
| Model: Y=Y0\*exp(k\*X). Y0 is the value when X (days) is zero. K is the rate constant. Tau is the time constant, reciprocal of K. Doubling-time was calculated as ln(2)/K. RSDR (Robust Standard Deviation of the Residuals). | Model: Y=Y0\*exp(k\*X). Y0 is the value when X (days) is zero. K is the rate constant. Tau is the time constant, reciprocal of K. Doubling-time was calculated as ln(2)/K. RSDR (Relative Standard Deviation). | | | | | | | |
1
