# Supplementary figures and images for: Modelling aggressive prostate cancers of young men in immune-competent mice, driven by isogenic Trp53 alterations and Pten loss
Source: Cell Death Dis. 2022 Sep 8;13(9):777. doi: 10.1038/s41419-022-05211-y (PMC9465983; doi:10.1038/s41419-022-05211-y)

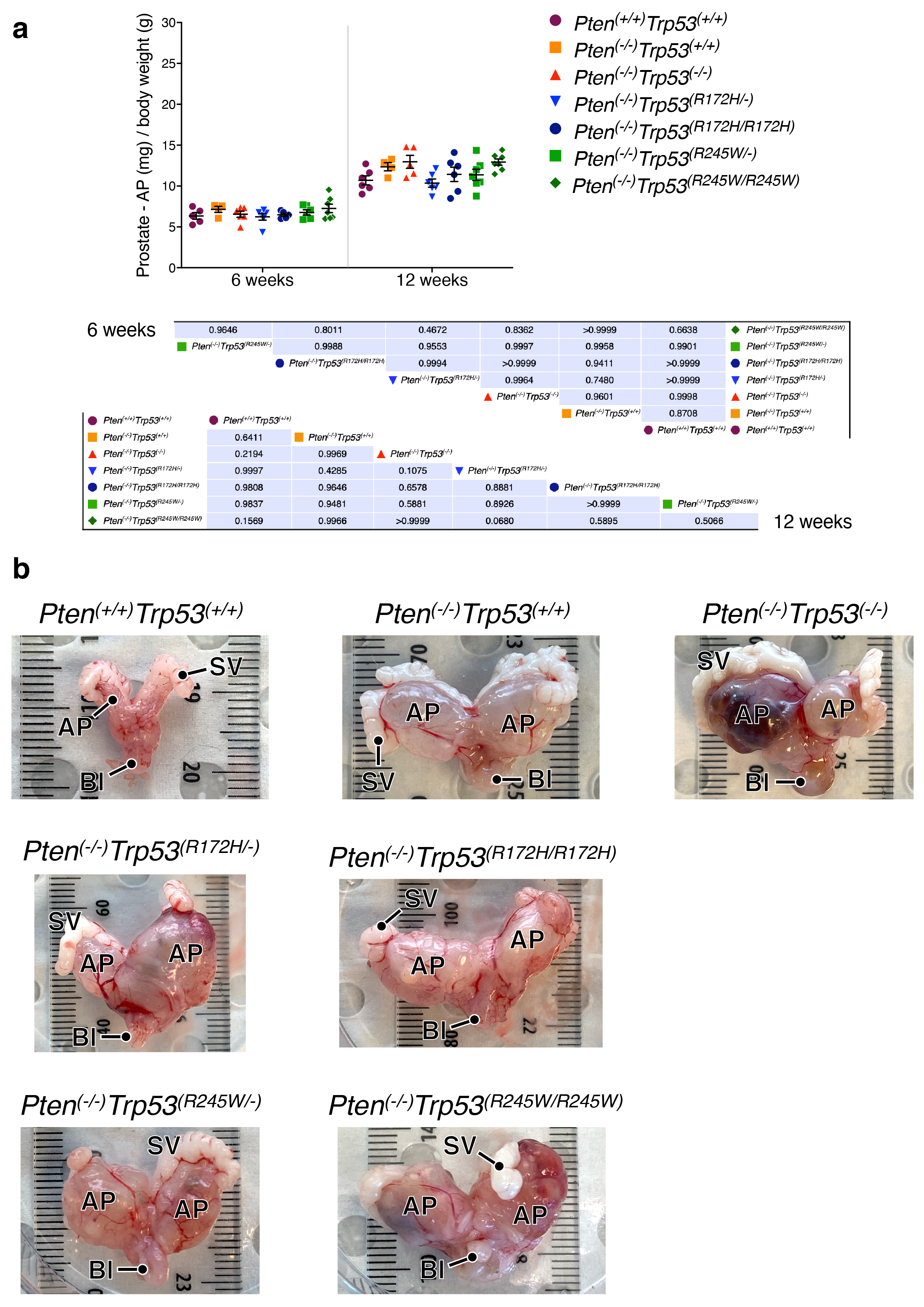

Supplement: Supplementary file 4 — Figure S1 [file 41419_2022_5211_MOESM4_ESM.png]

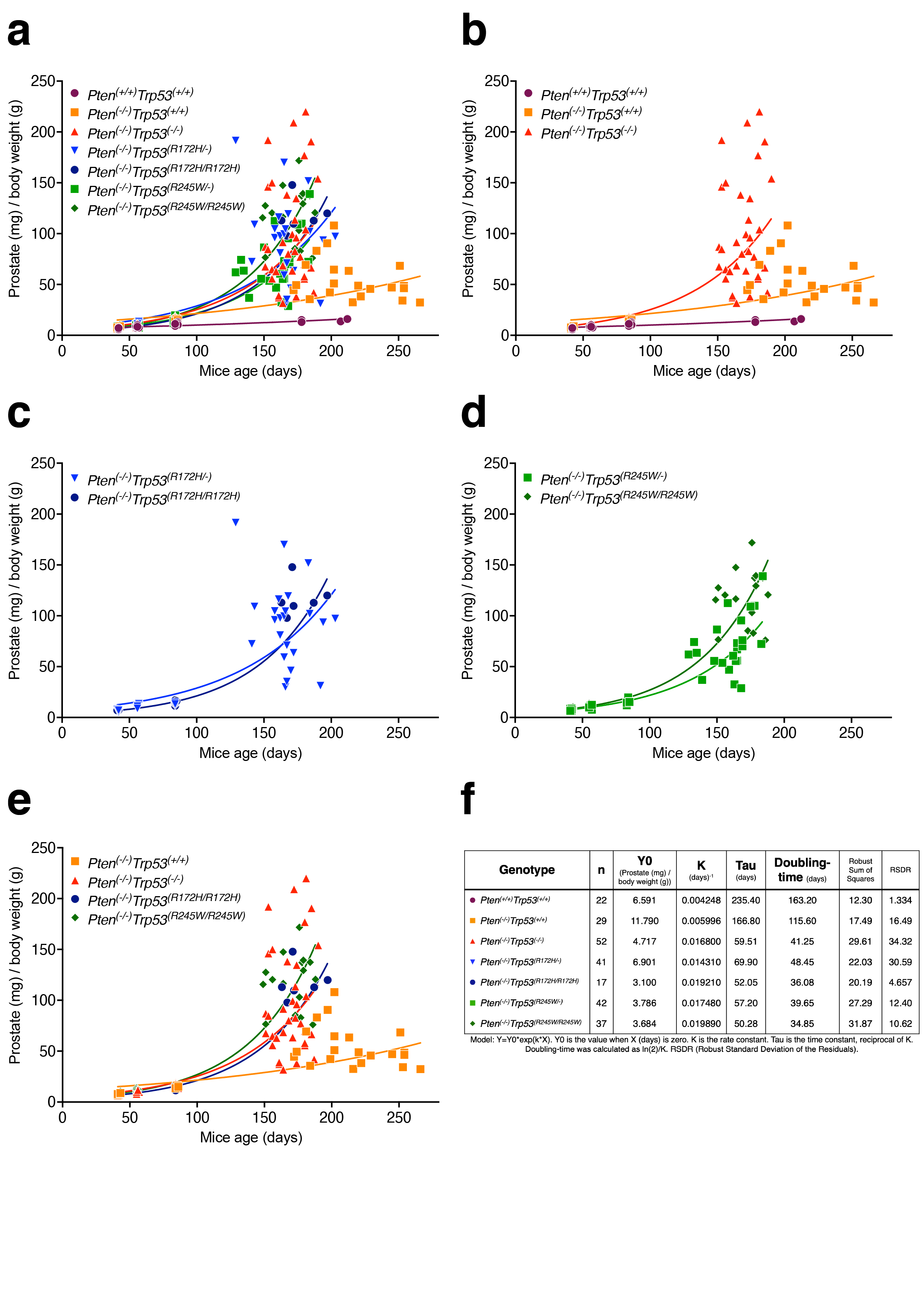

Supplement: Supplementary file 5 — Figure S2 [file 41419_2022_5211_MOESM5_ESM.png]

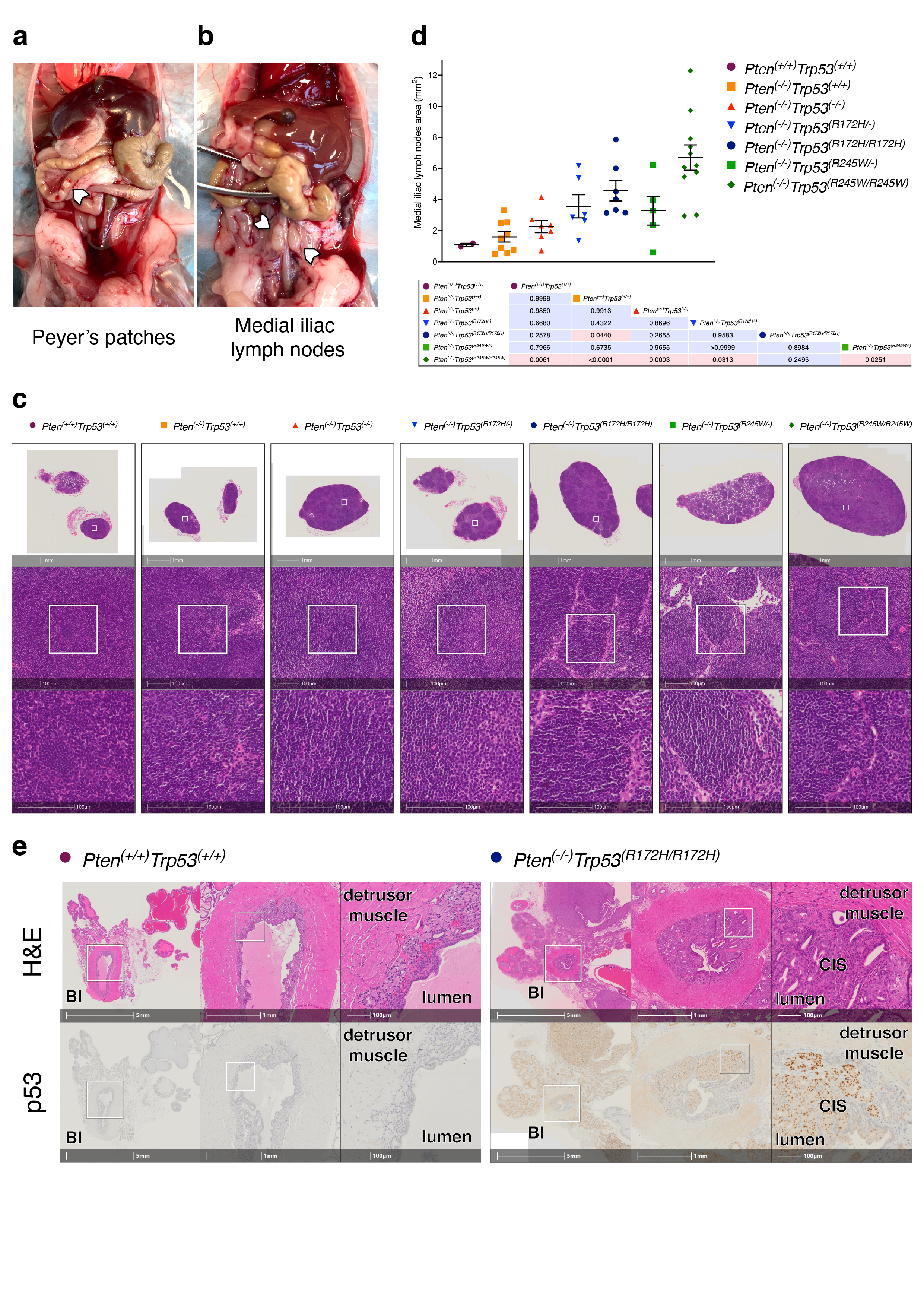

Supplement: Supplementary file 6 — Figure S3 [file 41419_2022_5211_MOESM6_ESM.png]

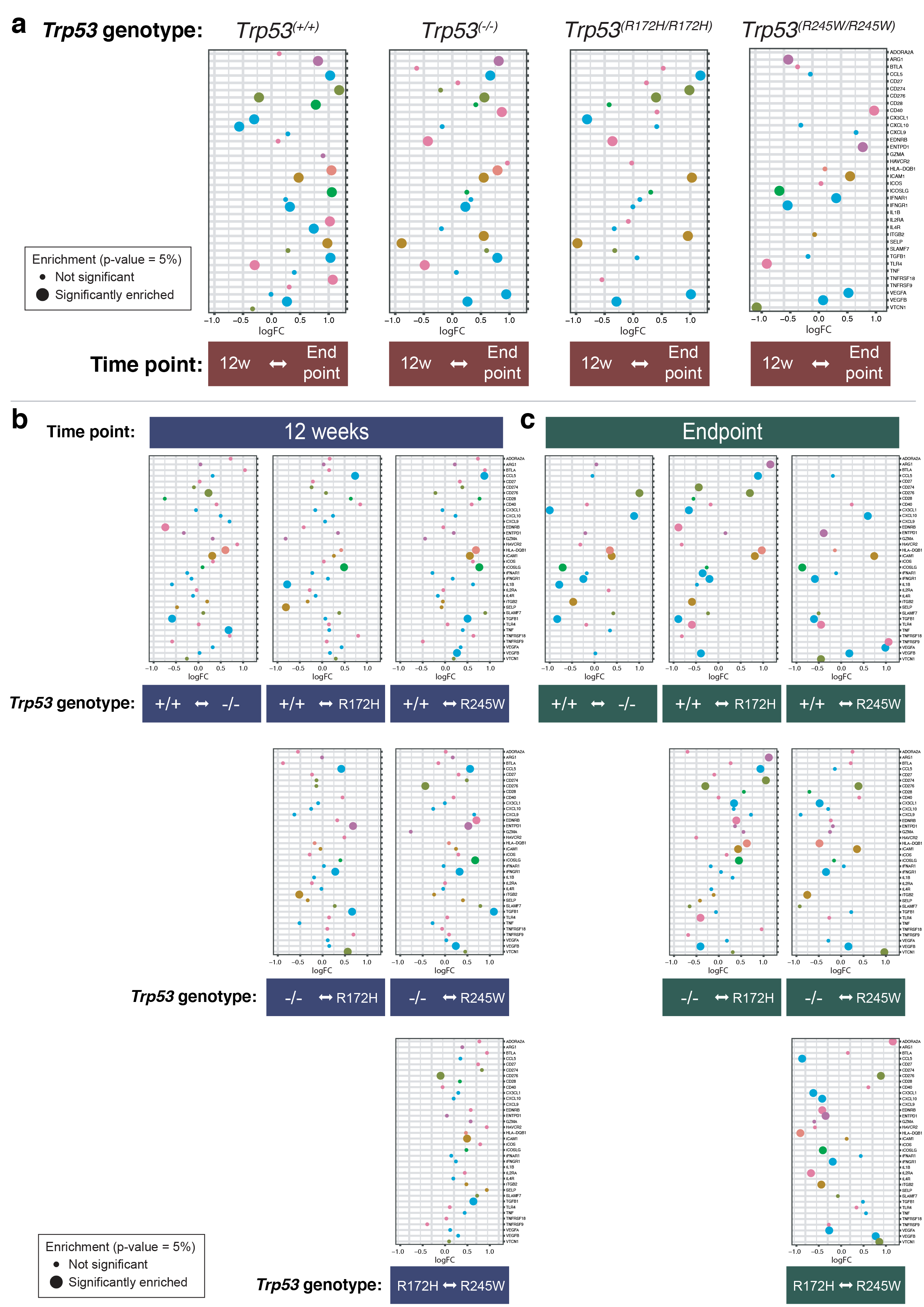

Supplement: Supplementary file 7 — Figure S4 [file 41419_2022_5211_MOESM7_ESM.png]

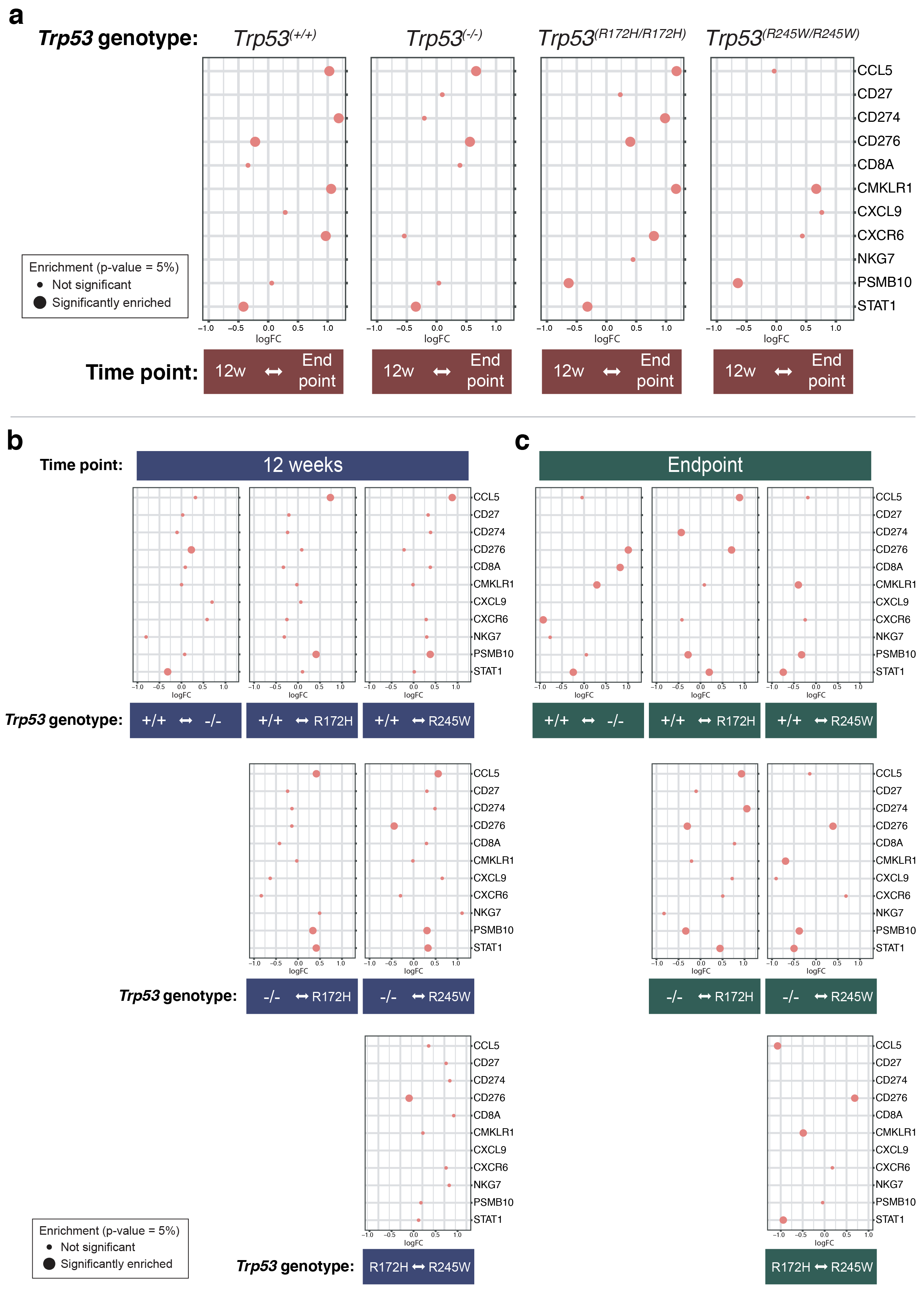

Supplement: Supplementary file 8 — Figure S5 [file 41419_2022_5211_MOESM8_ESM.png]
